# An elevated triglyceride-glucose index in the first-trimester predicts adverse pregnancy outcomes: a retrospective cohort study

**DOI:** 10.1007/s00404-025-07973-0

**Published:** 2025-02-26

**Authors:** Songhong Song, Qi Luo, Xinyang Zhong, Man Huang, Jinxiu Zhu

**Affiliations:** 1 Department of Preventive Medicine, Shantou University Medical College, Shantou, China; 2 Institute of Clinical Electrocardiography, First Affiliated Hospital of Shantou University Medical College, Shantou, 515041 Guangdong China; 3 Longgang Maternity and Child Institute of Shantou University Medical College, Shenzhen, 518172 Guangdong China; 4 School of Basic Medical Sciences, Jiamusi University, Jiamusi, 154007 China; 5 School of Pharmacy, Zunyi Medical University, Guizhou, 563006 China

**Keywords:** Triglyceride-glucose index, First-trimester, Pregnancy assessment, Maternal health, Adverse pregnancy outcomes

## Abstract

**Background:**

The relationship of the first-trimester triglyceride-glucose (TyG) index with GDM (gestational diabetes mellitus) and other adverse pregnancy outcomes has yet to be fully understood. This study aims to investigate the relationship between the first-trimester TyG index and the risk of adverse pregnancy outcomes in pregnant women.

**Methods:**

The data for the retrospective cohort study were derived from the Maternal and Child Health Hospital of Longgang District, Shenzhen, China. To calculate the TyG index, health indicators were measured in the early pregnancy period (<14 gestational weeks), including triglycerides and fasting blood glucose levels in pregnant women. Multivariable regression analysis and subgroup analysis were used to ascertain the independent association between the TyG index and the possibility of adverse pregnancy outcomes. Interaction analysis was performed to assess the potential heterogeneity of associations among subgroups. Nonlinear associations and the predictive value of the TyG index were explored using restricted cubic splines and receiver operating characteristic (ROC) curves. The discrimination and accuracy of the fully adjusted model were evaluated using calibration curves, Brier scores, and decision curve analysis (DCA). Mediation analysis was conducted to assess the impact of GDM (gestational diabetes mellitus) and PE (preeclampsia) as intermediaries on the risk of Preterm delivery.

**Results:**

The study included a cohort of 11,942 pregnant women, with an average TyG index of 8.36 ± 0.41. Logistic regression analysis showed that after adjusting for covariates, for each 1-unit increase in the TyG index, the risk of GDM increased by 2.21-fold, and this result was significantly different across all quartiles. Compared to the lowest quartile group, the highest TyG index group had the highest risk of PE (OR: 2.89; 95% CI 1.39 ~ 6.50), GH (gestational hypertension) (OR: 1.47; 95% CI 1.07 ~ 2.02), and Preterm delivery (OR: 1.75; 95% CI 1.21 ~ 2.56).The analysis of data stratification and interaction confirmed the validity of our study results. However, the analysis found no statistically significant association between the TyG index and low birth weight and macrosomia. GDM and PE were identified as partial mediating factors between TyG and the risk of preterm delivery, with variance contributions of 7.23% and 20.33%. The TyG index demonstrated the highest area under the curve (AUC) values in the ROC curves for GDM, PE, GH, and preterm delivery, with values of 0.61, 0.67, 0.58, and 0.56, respectively. The combination of the TyG index, maternal age, and pre-pregnancy body mass index predicted outcomes better than the TyG index alone (*p* < 0.01).After adjustment for confounders, the model showed good accuracy and net benefit in predicting adverse pregnancy outcomes, as supported by calibration curves, Brier scores, and decision curve analysis.

**Conclusion:**

An elevated first-trimester TyG index correlates with a heightened risk of GDM, PE, GH and Preterm delivery.The TyG index presents a promising tool for more effectively identifying populations at early risk for adverse pregnancy outcomes.

**Supplementary Information:**

The online version contains supplementary material available at 10.1007/s00404-025-07973-0.

## What does this study add to the clinical work

**Table:** 

This study demonstrates that an elevated first-trimester TyG index is significantly associated with an increased risk of GDM, PE, GH, and preterm delivery. The findings suggest that the TyG index can serve as a promising tool for more effectively identifying pregnant women at early risk of adverse outcomes, which has important implications for clinical practice and may help reduce screening expenditures.	

## Introduction

Gestational diabetes mellitus (GDM) is defined by glucose intolerance that either begins or is first recognized during pregnancy, typically diagnosed between the second and third trimesters, and is not attributable to pre-existing diabetes [1]. Medical interventions, including glucose monitoring, dietary and exercise adjustments, and pharmacological treatments, are commonly provided to women with overt diabetes or a diagnosis of GDM. Diagnostic confirmation of GDM usually occurs between 24 and 28 weeks of gestation by an oral glucose tolerance test (OGTT).However, as early pregnancy is a critical period for embryo and placenta development [2], this timing may be too late to prevent chronic adverse events.

Insulin resistance (IR) is characterized by a condition where target cells do not respond adequately to normal insulin levels, resulting in negative feedback causing excessive insulin secretion. Although physiological IR during pregnancy is beneficial for fetal growth and nutrient supply [3], if the degree of IR is significantly higher than that of normal pregnancy, it may lead to abnormal glucose metabolism, chronic inflammation, and oxidative stress, thereby creating an in utero environment enriched with glucose, lipids, amino acids, and other nutrients, leading to intrauterine growth restriction and fetal macrosomia, among other complications [4].

The triglyceride-glucose (TyG) index serves as a cost-effective and straightforward marker for IR. This index is calculated based on fasting plasma triglyceride and glucose levels [5]. In contrast to the hyperinsulinemic-euglycemic clamp (HEC), which is often regarded as the most reliable method for evaluating IR, the TyG index provides notable benefits by addressing concerns associated with time, cost, and technical feasibility. In addition, compared with the homeostasis model assessment of insulin resistance (HOMA-IR), the TyG index requires only the measurement of fasting blood glucose and triglyceride levels, without the need for insulin concentration measurement, avoiding the limitation of HOMA-IR in certain scenarios, such as in individuals receiving insulin therapy or lacking functional β-cells [6], and its predictive performance for IR is even superior to that of HOMA-IR [7].

Studies have demonstrated an association between it and an elevated risk of CVD within the general population [8, 9]. Previous studies have also shown that the TyG index can predict the risk of atherosclerosis, coronary artery calcification, and diabetes [10–12]. Nonetheless, detailed investigations into the association between the TyG index and the risk of pregnancy complications and adverse perinatal outcomes are relatively sparse. Our objective is to assess the overall influence of maternal TyG index in early pregnancy on pregnancy outcome risks via more comprehensive data analysis.

## Materials and methods

### Study population

The retrospective cohort study carried out at the Maternal and Child Health Hospital, Longgang District, Shenzhen, China, encompassing the period from May 2019 to April 2024. Pregnant women were retrospectively screened through the digital medical record system. In this cohort study, we included pregnant women who were 18 years or older, with a gestational age of less than 14 weeks, and who had completed fasting glucose and lipid profiling early in their pregnancy. Eligibility also required that they maintained a regular schedule of structured prenatal check-ups. Exclusion criteria for pregnant women included any of the following: (1) incomplete data [Missing outcome information or presence of extreme TyG index values (mean ± 3 standard deviations)] (2) multiple pregnancies (3) following the directives of the International Association of Diabetes and Pregnancy Study Groups, this study excluded individuals with a documented history of gestational diabetes, as confirmed by both medical record review and glucose tolerance assessment. (4) Pre-pregnancy hypertension (systolic blood pressure ≥140 mmHg or diastolic blood pressure ≥90 mmHg) (5) autoimmune diseases, malignant tumors. (6) Medication use. A total of 14,477 records meeting the inclusion criteria were retrieved from the hospital information system. Following the application of the exclusion criteria, the final analysis comprised a total of 11,942 people (Additional file 1: Fig. S1).

This study was approved by the Ethics Committee of the Maternal and Child Health Hospital of Longgang District, Shenzhen (LGFYKYXMLL-2024-88), and followed the principles of the Helsinki Declaration.

### Calculation of TyG index

The calculation formula for the TyG index is as follows: TyG index = Ln [fasting triglycerides (mg/dL) × fasting blood glucose (mg/dL)/2] [13]. This equation describes the logarithmic transformation of the product of fasting triglycerides and glucose levels, divided by 2.

In this study, we treated the TyG index as a continuous variable. Subsequently, given that quartile analysis can preliminarily identify the relationship between different levels of TyG index and adverse pregnancy outcomes, it can provide a basis for the determination of further clinically relevant cutoff values. Therefore, we divided the TyG index into quartiles for further analysis. Values below the 25^th^ percentile were defined as Q1, values between the 25^th^ and 50^th^ percentiles were defined as Q2, values between the 50^th^ and 75^th^ percentiles were defined as Q3, and values equal to or above the 75^th^ percentile were defined as Q4.

### Adverse pregnancy outcomes

Diagnostic information related to gestational diabetes mellitus, gestational hypertension (GH), and preeclampsia (PE) was extracted from the medical records of all enrolled pregnant women and electronically recorded. All enrolled women underwent a diagnostic 75-gram OGTT between 24 and 28 weeks of gestation. The diagnosis of GDM was based on the IADPSG criteria for OGTT (meeting or exceeding any of the OGTT values: fasting ≥ 5.1 mM; 1 hour ≥ 10.0 mM; 2 hours ≥ 8.5 mM) [14]. GH is defined as new-onset hypertension occurring twice, with each instance at least 4 hours apart, and a blood pressure reading of ≥140/90, as established by the ACOG in 2019. PE is characterized by hypertension accompanied by proteinuria or other manifestations of end-organ damage, such as thrombocytopenia with platelet counts below 100×10^9^/L, renal impairment indicated by a serum creatinine level exceeding 1.1 mg/dL or a twofold increase from the baseline, hepatic dysfunction with transaminase levels more than double the normal range, pulmonary edema, and new-onset headaches that are refractory to treatment or accompanied by visual disturbances [15]. Neonatal outcome information was obtained from birth records, including low birth weight (LBW) (defined as newborns weighing less than 2500 grams at birth), macrosomia (birth weight over 4000 grams), and Preterm delivery (delivery between 28 and 37 weeks of gestation) [16].

### Covariates

Beyond the primary variables, this study also took into account various other covariates that are recognized for their potential influence on the assessment of outcomes. Maternal characteristics and pregnancy outcomes data were collected from the hospital's information system, also as information pertaining to laboratory testing. Demographic and medical information included the age of the pregnant women, level of education, pre-pregnancy weight, height, pre-pregnancy body mass index (BMI), gravidity, parity, diastolic blood pressure (DBP) and systolic blood pressure (SBP). A maternal pre-pregnancy BMI was calculated by dividing the weight in kilogrammes before pregnancy by the square of the height in meters. It was then categorised as follows: malnourished (BMI < 18.5 kg/m^2^), within the normal weight range (18.5≤BMI < 25.0 kg/m^2^), or overweight or obese (BMI ≥ 25.0 kg/m^2^) [17]. Following a minimum fasting period of 8 hours, blood samples were obtained from all participants for the purpose of analyzing biomarkers such as fasting plasma glucose (FPG, mg/dL), triglycerides (TG, mg/dL), total cholesterol (TC, mg/dL), low-density lipoprotein cholesterol (LDL-C, mg/dL), high-density lipoprotein cholesterol (HDL-C, mg/dL), glycated hemoglobin (HbA1c, g/L), total protein (TP, g/L), and albumin (ALB, g/L).

### Statistical analysis

Continuous variables are reported as the mean ± standard deviation, whereas categorical variables are depicted in terms of frequencies and percentages. There is no consensus on a specific cutoff value for the TyG index to diagnose IR. Therefore, based on previous research, all included participants were divided into four equal groups based on the TyG index [18]. This cutoff value was followed in further statistical analyses. Analysis of variance and chi-square tests were conducted to analyze differences between groups.

We employed a multivariate logistic regression approach to calculate the odds ratios (OR) together with their associated 95% confidence intervals (CI), examining the correlation between various TyG index quartiles and the risk of adverse pregnancy outcomes. The first quartile was used as the reference group. We controlled for confounding bias by adjusting for different covariates. To address multicollinearity, we excluded variables with a variance inflation factor (VIF) exceeding 5 before selecting the remaining variables. Model 1 served as the unadjusted baseline, Model 2 incorporated adjustments for Age, Education, Pre-pregnancy BMI, Gravidity, Parity, and gestational week at examination。Model 3 further refined the analysis with additional adjustments for Age, Education, Pre-pregnancy BMI, Gravidity, Parity, gestational week at the examination, SBP, DBP, TC, LDL, HDL, HbA1c, TP and ALB. In the sensitivity analyses, the TyG index was categorized into quartiles to determine the robustness of the findings and to assess the risk of adverse pregnancy outcomes associated with each quartile. Moreover, restricted cubic splines (RCS) can flexibly model and visualize the relationships between variables. Even if the relationship is ultimately found to be linear, RCS can help confirm this linearity and rule out the possibility of a nonlinear relationship. Therefore, we employed RCS to explore the potential nonlinear relationship between the TyG index and the likelihood of adverse pregnancy outcomes.Stratified analyses of the TyG index and the risk of adverse pregnancy outcomes were conducted based on maternal age (≤29y/30-34y/≥35y), pre-pregnancy BMI (Underweight/Normal weight/Overweight&Obese), Gravidity (≤2/>2), parity (0/≥1), and education (≤12y/>12y). Interaction analysis was performed to assess the potential heterogeneity of associations among subgroups.The predictive value of TyG for various adverse pregnancy outcomes was assessed using the ‘pROC’ R package, and the predictive capabilities of the “TyG index” and the “TyG index combined with maternal age and pre-pregnancy BMI” for adverse pregnancy outcomes were also compared. The discrimination and accuracy of the fully adjusted model were evaluated using calibration curves, Brier scores, and decision curve analysis (DCA). To evaluate whether GDM, PE, and GH act as intermediaries between TyG and preterm delivery, we employed a mediation model of path analysis. We first established a direct effect model of TyG on the risk of preterm delivery, and subsequently, GDM, PE, and GH were included in the model as mediating variables, with the influence of TyG on these mediating variables as well as their impact on the risk of preterm delivery being assessed separately.A two-sided P value of less than 0.05 was employed to determine statistical significance. The R Foundation makes available R version 4.4.1 at http://www.R-project.org, which was employed to conduct the analyses.

## Results

### Baseline characteristics

The baseline characteristics of the study’s participants are shown in Table 1, classified by quartiles of the TyG index. It included a cohort of 11,942 individuals with an average age of 30.12 ± 4.09 years. The TyG index for the participants had an average value of 8.36 ± 0.41. The TyG index quartile ranges were delineated as 6.14–8.08 for the first, 8.09–8.33 for the second, 8.34–8.61 for the third, and 8.62–10.72 for the fourth quartile. The overall average prevalence of GDM was 15.4%. A correlation was observed between higher TyG index quartiles and increased prevalence of GDM: 10.3% for the first quartile, 12.8% for the second, 15.4% for the third, and 23.2% for the fourth. This trend was also observed for the prevalence of GH and PE, as well as the incidence of low birth weight, which escalated with higher TyG index quartiles: for GH, the prevalence rates were 3.0%, 3.2%, 3.6%, and 4.2%; for PE, the rates were 0.3%, 0.5%, 0.7%, and 1.6%; and for low birth weight, the rates were 1.0%, 1.3%, 1.4%, and 1.7%. Higher TyG index among participants was associated with a lower level of education. Pre-pregnancy BMI, SBP, DBP, gravidity, parity, and maternal age all significantly increased in tandem with the TyG index. Similarly, FPG, TG, TC, HbA1c, TC, and LDL showed significant positive correlations with the TyG index (*P* < 0.001). In contrast, ALB decreased with the increase in the TyG index (*P* < 0.001). The levels of HDL rose initially and subsequently declined with an increase in the TyG index, a pattern that was highly significant (*P* < 0.001). Conversely, TP levels did not vary significantly when analyzed across the quartiles of the TyG index.

**Table 1:** Maternal characteristics and pregnancy complications baseline data of the study population

Characteristics	Quartiles of TyG index				Overall	*P* value
	Q1 (n = 2986)	Q2 (n = 2985)	Q3 (n = 2986)	Q4 (n = 2985)	Total (n = 11942)	
	(6.14–8.08)	(8.09–8.33)	(8.34–8.61)	(8.62–10.72)		
TyG index	7.88 ± 0.18	8.21 ± 0.07	8.47 ± 0.08	8.89 ± 0.26	8.36 ± 0.41	**<0.001**
Age, years	29.21 ± 3.77	29.85 ± 3.94	30.30 ± 4.08	31.12 ± 4.33	30.12 ± 4.09	**<0.001**
Education, years						**<0.001**
9	262 (8.8)	276 (9.2)	357 (12.0)	407 (13.6)	1302 (10.9)	
10–12	179 (6.0)	242 (8.1)	243 (8.1)	303 (10.2)	967 (8.1)	
13–15	1224 (41.0)	1204 (40.3)	1234 (41.3)	1245 (41.7)	4907 (41.1)	
≥16	1321 (44.2)	1263 (42.3)	1152 (38.6)	1030 (34.5)	4766 (39.9)	
Pre-pregnancy BMI, kg/m^2^	20.45 ± 2.57	21.03 ± 2.79	21.64 ± 2.93	22.80 ± 3.35	21.48 ± 3.05	**<0.001**
Gravidity, n (%)						**<0.001**
1	1573 (52.7)	1393 (46.7)	1187 (39.8)	919 (30.8)	5072 (42.5)	
2	840 (28.1)	869 (29.1)	902 (30.2)	980 (32.8)	3591 (30.1)	
3	343 (11.5)	415 (13.9)	519 (17.4)	598 (20.0)	1875 (15.7)	
≥4	230 (7.7)	308 (10.3)	378 (12.7)	488 (16.3)	1404 (11.8)	
Parity, n (%)						**<0.001**
0	1968 (65.9)	1797 (60.2)	1550 (51.9)	1265 (42.4)	6580 (55.1)	
1	830 (27.8)	936 (31.4)	1135 (38.0)	1333 (44.7)	4234 (35.5)	
≥2	188 (6.3)	252 (8.4)	301 (10.1)	387 (13.0)	1128 (9.4)	
Gestational week at examination, weeks	8.46 ± 1.70	8.92 ± 1.78	9.46 ± 1.85	9.90 ± 1.90	9.18 ± 1.89	**<0.001**
SBP, mmHg	109.09 ± 10.84	110.30 ± 10.82	112.06 ± 11.03	113.52 ± 11.35	111.25 ± 11.14	**<0.001**
DBP, mmHg	65.04 ± 8.34	66.64 ± 8.52	68.00 ± 8.47	69.19 ± 8.72	67.22 ± 8.65	**<0.001**
FPG, mg/dL	81.69 ± 7.07	83.09 ± 7.22	84.11 ± 7.71	87.74 ± 12.74	84.16 ± 9.27	**<0.001**
TG, mg/dL	65.93 ± 11.17	89.78 ± 9.57	114.40 ± 13.03	173.56 ± 55.98	110.91 ± 49.81	**<0.001**
HbAlc, %[mmol/mol]	4.88 ± 0.39 [53.33 ± 4.26]	4.93 ± 0.35 [53.88 ± 3.83]	4.95 ± 0.37 [54.10 ± 4.04]	5.04 ± 0.45 [55.08 ± 4.92]	4.95 ± 0.40 [54.10 ± 4.37l]	**<0.001**
TC, mmol/L	4.01 ± 0.60	4.21 ± 0.63	4.41 ± 0.65	4.61 ± 0.73	4.31 ± 0.69	**<0.001**
HDL, mmol/L	1.53 ± 0.28	1.55 ± 0.28	1.55 ± 0.29	1.47 ± 0.29	1.52 ± 0.29	**<0.001**
LDL, mmol/L	1.97 ± 0.49	2.13 ± 0.51	2.28 ± 0.52	2.47 ± 0.61	2.21 ± 0.56	**<0.001**
TP, g/L	66.05 ± 3.62	66.05 ± 3.68	66.10 ± 3.76	66.26 ± 3.68	66.12 ± 3.69	0.082
ALB, g/L	38.18 ± 2.47	37.96 ± 2.62	37.88 ± 2.62	37.76 ± 2.56	37.94 ± 2.57	**<0.001**
Macrosomia, n (%)	93 (3.1)	77 (2.6)	100 (3.3)	108 (3.6)	378 (3.2)	0.128
Low birth weight, n (%)	31 (1.0)	39 (1.3)	41 (1.4)	52 (1.7)	163 (1.4)	0.133
Preterm delivery, n (%)	64 (2.1)	58 (1.9)	78 (2.6)	106 (3.6)	306 (2.6)	**<0.001**
GDM, n (%)	309 (10.3)	382 (12.8)	459 (15.4)	692 (23.2)	1842 (15.4)	**<0.001**
GH, n (%)	91 (3.0)	95 (3.2)	108 (3.6)	124 (4.2)	418 (3.5)	0.086
PE, n (%)	10 (0.3)	15 (0.5)	20 (0.7)	49 (1.6)	94 (0.8)	**<0.001**

Significant findings in bold values (*P* < 0.05)

Categorical data are presented as n (%). Continuous variables are presented as means ± SD

*TyG index* triglyceride-glucose index, *Q* quartiles, *BMI* body mass index, *SBP* systolic blood pressure, *DBP* diastolic blood pressure, *HbA1c* hemoglobin type A1C, *FPG* fasting plasma glucose, *TG* triglyceride, *HDL* high-density lipoprotein, *LDL* low density lipoprotein, *TP* Total Protein, *ALB* albumin, *GDM* gestational diabetes mellitus, *GH* Gestational Hypertension, *PE* preeclampsia

The findings reveal a positive correlation between advancing maternal age and elevated levels of TG, TC, FPG, as well as an augmented TyG index and heightened prevalence of GDM and Preterm delivery (Additional file 1: Table S1).

### Relationship of TyG index with the risk of adverse pregnancy outcomes

In the study of GDM, a significant correlation was identified between elevated TyG index values and the risk of GDM (Additional file 1: Table S2). In the comprehensively adjusted model (Model 3), the positive association was found to be consistently robust (OR = 2.21, 95% CI: 1.90–2.57, *P* < 0.001). Additionally, we performed a sensitivity analysis, categorizing the TyG index into quartiles from its continuous form. The ORs and their corresponding 95% CIs for the TyG index quartiles, ranging from the lowest to the highest after multivariate adjustment, were as follows: 1.00 (reference), 1.20 (1.02–1.41), 1.44 (1.23–1.70), and 2.14 (1.81–2.54). Regarding the risk of PE, GH, and Preterm delivery (Additional file 1: Tables S3–S5), We noted a notable positive correlation between an elevated TyG index and the increased risk of PE (Model 3: OR = 2.53, 95% CI 1.43–4.44, *P* < 0.001), GH (Model 3: OR = 1.49, 95% CI: 1.12–1.97,* P* = 0.006), and Preterm delivery (Model 3: OR = 1.78, 95% CI 1.27–2.48, *P* < 0.001). In the sensitivity analysis, the adjusted OR for the quartiles of PE, GH, and Preterm delivery (reference quartile 1) were 2.89 (95% CI 1.39–6.50), 1.47 (95% CI 1.07–2.02), and 1.75 (95% CI 1.21–2.56), respectively, indicating a consistent positive correlation between an increased TyG index and the risk of PE, GH and Preterm delivery, which is statistically significant. Within the scope of our investigation, no substantial association was identified between the TyG index and the occurrence of macrosomia and low birth weight (Additional file 1: Tables S6 and S7).

### RCS analysis

**Fig. 1:**
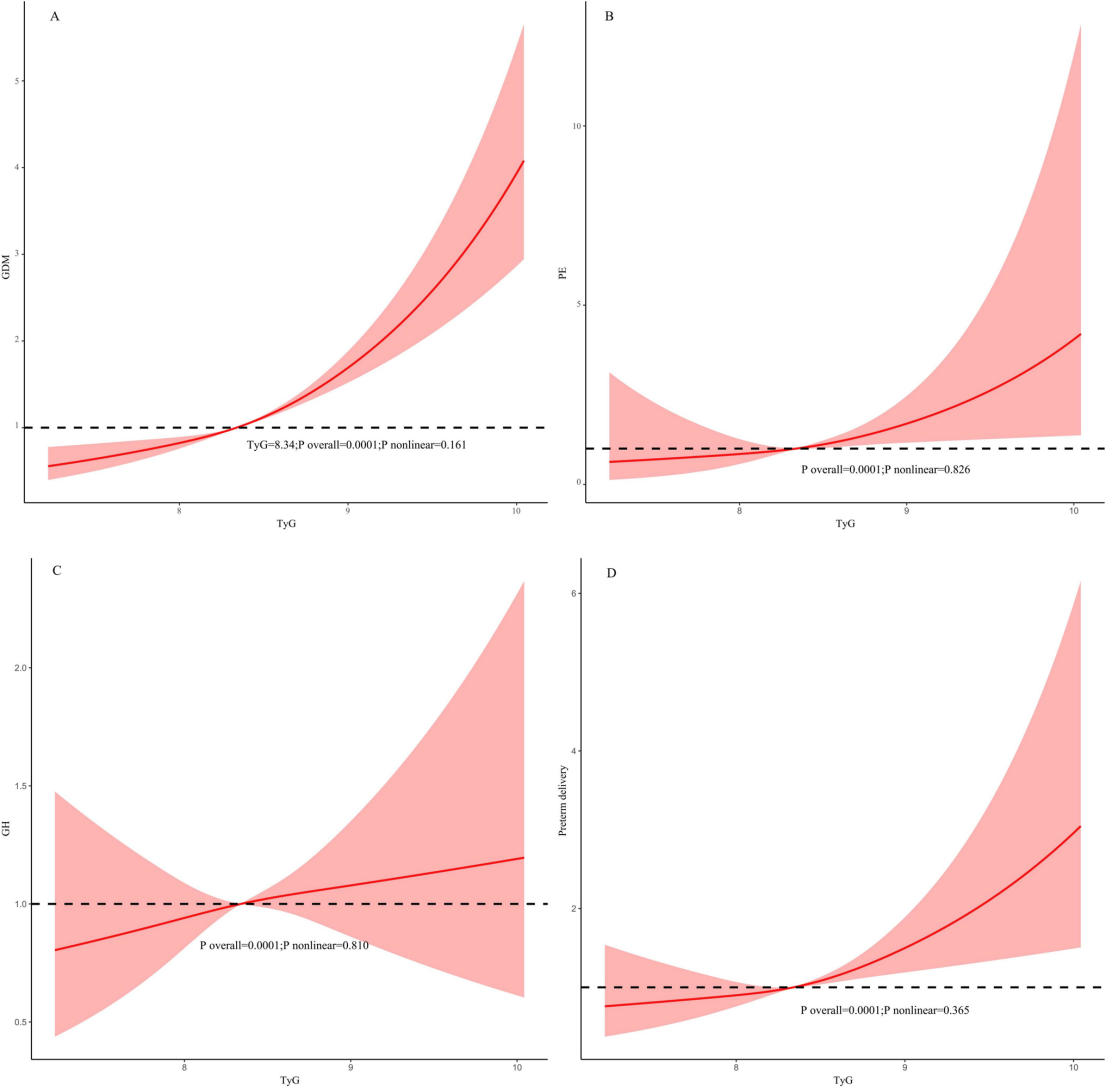
The restricted cubic spline analysis between the TyG index and the risk of GDM (**A**), PE(B), GH (**C**), and Preterm delivery (**D**). *TyG index* triglyceride-glucose index, *GDM* gestational diabetes mellitus, *GH* Gestational Hypertension, *PE* preeclampsia

We employed RCS curves to assess the possible non-linear associations between the TyG index and the risks of GDM, PE, GH, and preterm delivery. Our study results indicate that no significant non-linear correlation exists between the TyG index and the occurrence of GDM (overall *P*-value = 0.0001, nonlinear *P*-value = 0.161), PE (overall *P*-value = 0.0001, *P*-value for nonlinearity = 0.826), GH (overall *P*-value = 0.0001, *P*-value for nonlinearity = 0.810), and preterm delivery (overall *P*-value = 0.0001, nonlinear *P***-**value = 0.365) (Fig. 1).

### Subgroup analysis

**Fig. 2:**
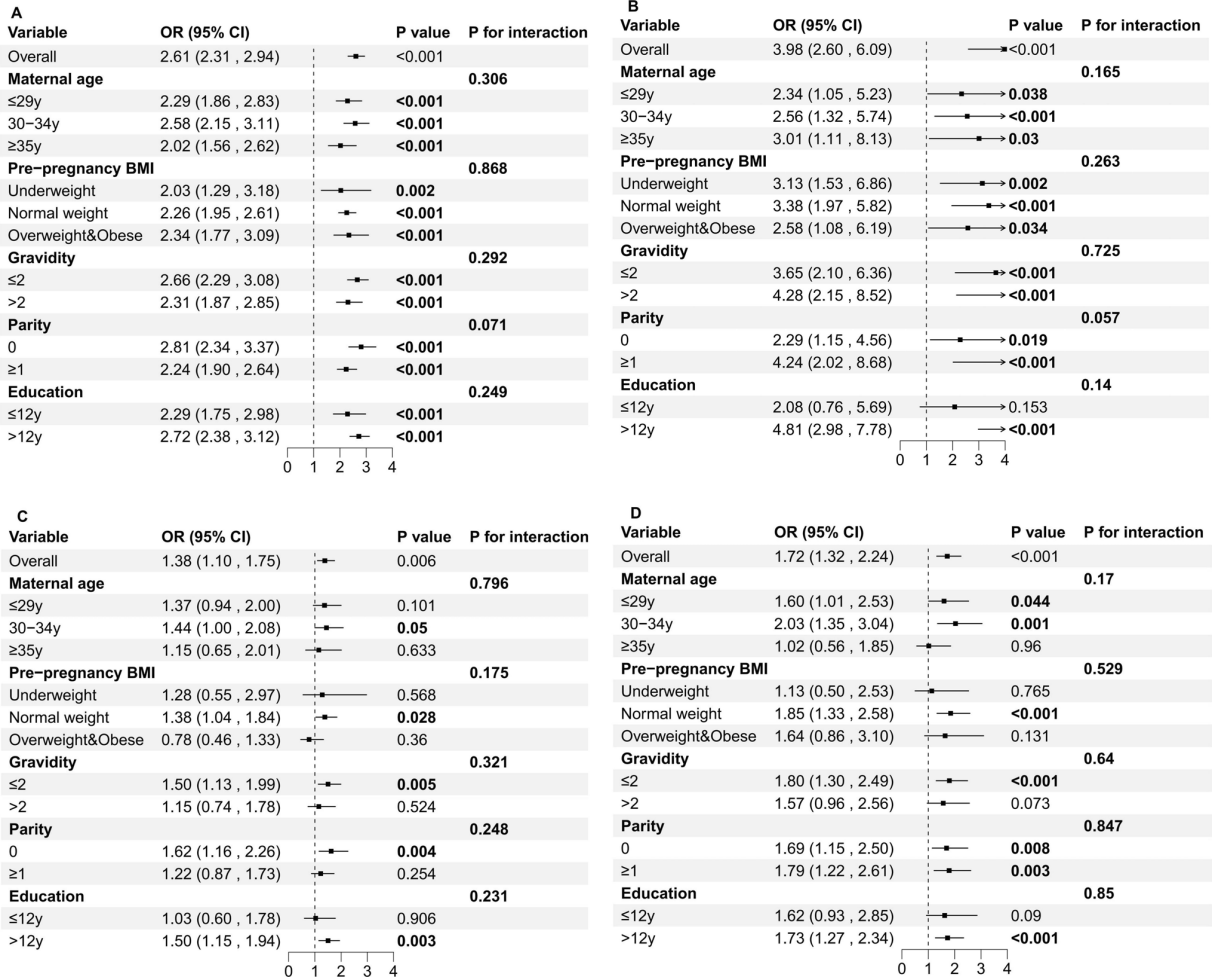
Subgroup analysis for the association between the TyG index and the risk of GDM (**A**), PE (**B**), GH (**C**), and Preterm delivery (**D**). *TyG index* triglyceride-glucose index, *GDM* gestational diabetes mellitus, *GH* Gestational Hypertension, *PE* preeclampsia

Subgroup analyses and interaction tests were conducted to assess the consistency of the correlation between the TyG index and the risk of GDM, PE, GH, and preterm delivery among different demographic groupings. These subgroups included maternal age, pre-pregnancy BMI, gravidity, parity, and level of education. The TyG index demonstrated a significant correlation with an elevated risk of GDM, thereby reinforcing the reliability of the findings (Fig. 2A). Regarding the risk of PE, significant associations were observed in all other subgroups except for the one with an education level of less than 12 years (Fig. 2B). However, the strong correlation between the TyG index and the occurrence of GH and preterm delivery was more likely to occur in pregnant women aged 30–34, with a pre-pregnancy BMI categorized as Normal weight, gravidity ≤2, nulliparous, and an education level greater than 12 years (Fig. 2C, D). Interaction terms were employed to evaluate the heterogeneity across various subgroups, and no statistically significant correlation with the *P*-values for interaction was observed for the risk of GDM, PE, GH and preterm delivery, indicating that this association is not dependent on age, pre-pregnancy BMI, gravidity, parity, and level of education (Fig. 2).

### The association of GDM, PE, GH, Preterm delivery, Macrosomia, and Low birth weight on TyG index levels

Furthermore, we conducted a detailed analysis to examine the variations in TyG index levels associated with various adverse pregnancy outcomes (Additional file 1: Table S8). In the comprehensively adjusted Model 3, In the comprehensively adjusted Model 3, the TyG index increments were 0.082 (β: 0.082, 95% CI 0.064–0.097), 0.074 (β: 0.074, 95% CI 0.043–0.178), and 0.038 (β: 0.038, 95% CI 0.032–0.107) units for those with GDM, PE, and Preterm delivery, respectively, surpassing the levels observed in pregnant women without these conditions. In contrast, no significant changes in the TyG index were noted among pregnant women experiencing GH, Macrosomia, and Low birth weight. Additionally, we assessed the influence of age on TyG index variations in the subgroups of participants affected by GDM, PE, and Preterm delivery (Additional file 1: Table S9). Our findings revealed that, upon accounting for confounding variables, the increment in the TyG index varied significantly across subgroups, particularly in the context of GDM. However, the significance of the increase in the TyG index in the PE and Preterm delivery groups was more likely to occur in the subgroups aged ≤24 years, ≥35 years, and ≤24 years, 25–29 years, respectively. We observed no statistically significant interaction *p*-values using interaction terms to assess heterogeneity between each subgroup, suggesting that the association is independent of age.

### Mediation and moderation model for the association between TyG and Preterm delivery

**Fig. 3:**
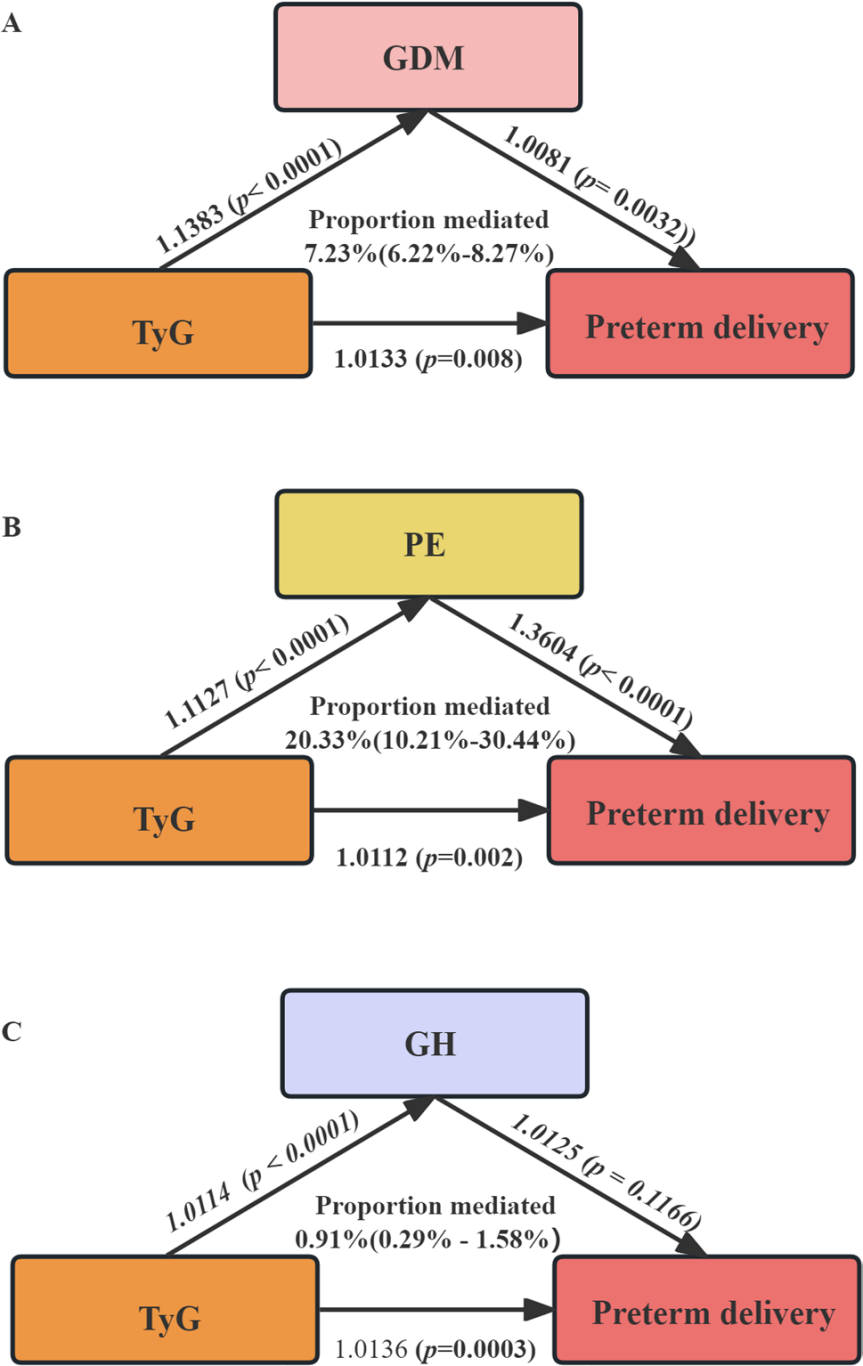
The mediation and moderation models for Preterm delivery utilizes GDM (**A**), PE (**B**) or GH (**C**) as mediator. *GDM* gestational diabetes mellitus, *GH* Gestational Hypertension, *PE* preeclampsia

This study explored the role of GDM, PE, and GH as intermediaries between TyG and preterm delivery. The results indicated significant associations between TyG and the risk of GDM (OR: 1.1383, 95% CI 1.1287–1.1479, *P* < 0.0001), PE (OR: 1.1127, 95% CI 1.0107–1.0147, *P* < 0.0001), and GH (OR: 1.0114, 95% CI 1.0072–1.0155, *P* < 0.0001). Additionally, GDM (OR: 1.0081, 95% CI 1.0040–1.0121, *P*= 0.0032), PE (OR: 1.3604, 95% CI 1.2403–1.2810, *P* < 0.0001) are both significantly associated with the risk of preterm delivery. After controlling for the exposure variable, the impact of the intermediary variable GH on the risk of preterm delivery showed no statistical difference (OR: 1.0125, 95% CI 1.0045–1.0204, *P* = 0.1166). The risk of preterm delivery, 7.23% can be attributed to TyG’s intermediary role in GDM (95% CI 6.22%–18.83%), and 20.33% can be attributed to its intermediary role in PE (95% CI 10.21%–30.34) (Fig. 3).

### The predictive value of the TyG index for the risk of GDM, PE, GH, and preterm delivery

**Fig. 4:**
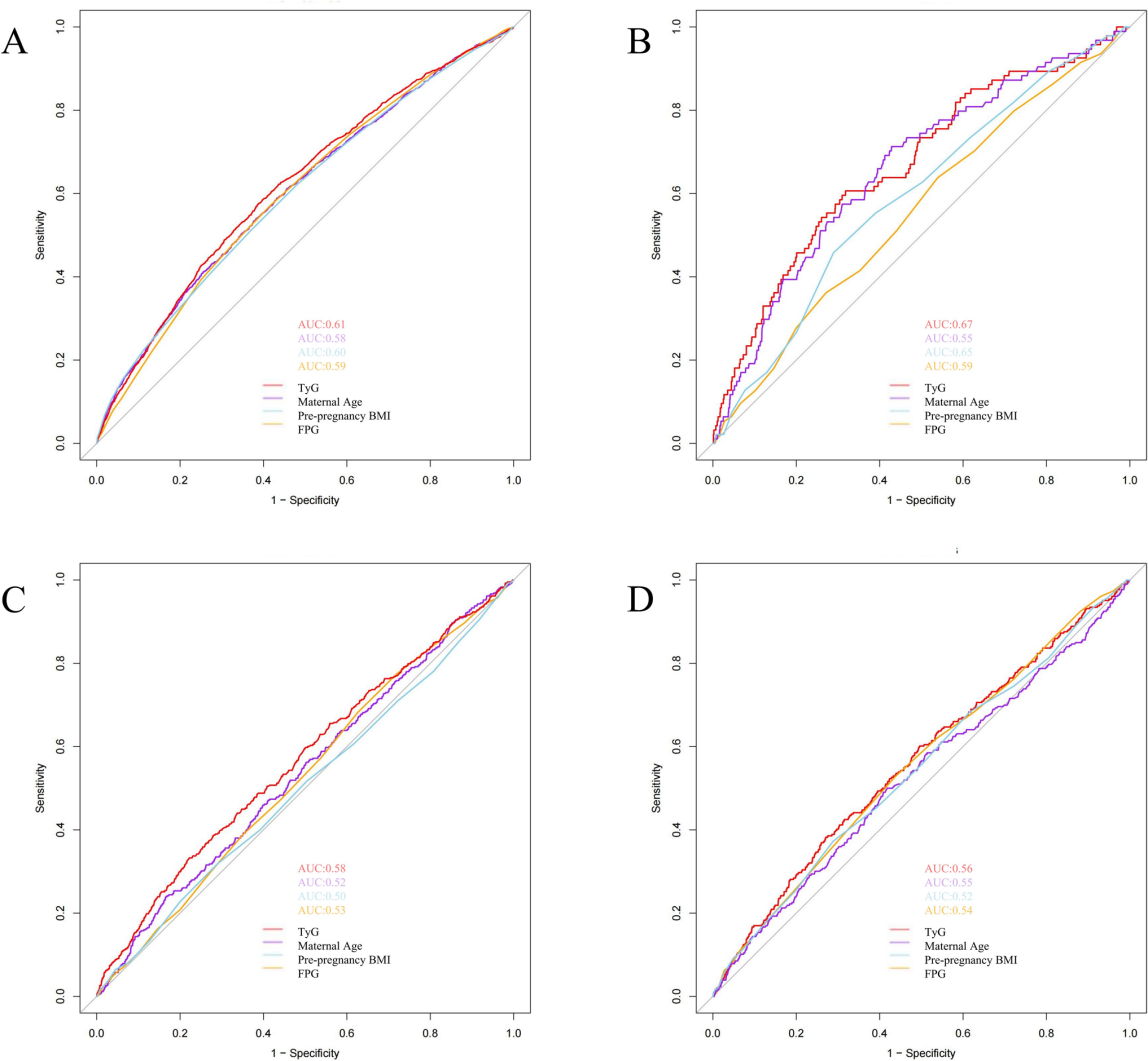
ROC curves of TyG index, maternal age, pre-pregnancy BMI and FPG for predicting the development of GDM (**A**), PE (**B**), GH (**C**), and Preterm delivery(**D**). *TyG index* triglyceride-glucose index, *GDM* gestational diabetes mellitus, *GH* Gestational Hypertension, *PE* preeclampsia

The independent predictive capabilities of the TyG index, maternal age, pre-pregnancy BMI, and FPG for GDM, PE, GH, and preterm delivery were evaluated using ROC curves and AUC values. Using the DeLong method, it was found that the TyG index outperformed other indices in predicting these adverse outcomes (*P* < 0.05) (Fig. 4). Additionally, ROC curves were generated to compare the predictive ability of the TyG index combined with maternal age and pre-pregnancy BMI for GDM, PE, GH, and preterm delivery. It was found that the combined prediction was superior to the predictive ability of the TyG index alone (*P* < 0.05) (Additional file 1: Fig. S2).After adjusting for all confounding factors, the calibration curves and Brier scores indicated that the model demonstrated good accuracy in predicting adverse pregnancy outcomes. In addition, decision curve analysis (DCA) was performed to assess the clinical utility of the model, which demonstrated that the model had good overall net benefit within the most reasonable threshold probabilities (Additional file 1: Fig. S3 and Additional file 1: Fig. S4).

## Discussion

This retrospective cohort study, including 11,942 pregnant women, was designed to investigate the association between the TyG index in the first trimester and the risk of adverse pregnancy outcomes (particularly GDM).Our research determined that an elevated TyG index is significantly and independently linked to a heightened risk of GDM, PE, GH, and preterm delivery. A linear correlation exists between the TyG index and the risk of GDM, PE, GH, and preterm delivery. Mediation analysis indicated that GDM and PE mediated the relationship between the TyG index and the risk of preterm delivery. Compared with maternal age, pre-pregnancy BMI, and FPG, the TyG index demonstrated higher predictive accuracy in forecasting the risks of GDM, PE, GH, and preterm delivery.Our results are robust for sensitivity and stratified analyses.

Insulin secretion or sensitivity impairment is generally considered the primary pathophysiology of various conditions. Pregnant women with overt IR are at a higher risk of adverse pregnancy outcomes [19].GDM is a prevalent condition characterized by glucose intolerance that is first recognized during pregnancy [20]. The association between IR and the increased risk of GDM has been extensively studied, revealing significant correlations [21]. IR itself is a key mechanism that develops during pregnancy. This physiological change is essential to ensure that the fetus has an adequate supply of glucose, but it can become excessive, leading to gestational diabetes. The body's inability to effectively utilize insulin leads to an insufficient insulin supply to meet the increased demands of pregnancy, which is associated with chronic IR present before pregnancy [22]. Chronic β-cell dysfunction is another significant factor leading to GDM. This dysfunction indicates that pancreatic β-cells gradually become incapable of producing sufficient insulin to counterbalance the rise in IR. In most GDM patients, this β-cell dysfunction occurs against the backdrop of chronic IR, and the additional IR that occurs during pregnancy exacerbates this condition, leading to the development of GDM [23].

PE, a complex and critical form of hypertension during pregnancy, affects an estimated 2%–8% of pregnancies across the globe [24]. The pathophysiological mechanism linking IR to preeclampsia involves several interconnected processes. IR, a condition where cells fail to respond effectively to insulin, is associated with elevated levels of inflammatory markers such as leptin and TNF-alpha, which are also altered in PE [25]. This resistance can lead to endothelial dysfunction, characterized by impaired vasodilation and increased vascular resistance, which is a hallmark of PE [26]. Furthermore, placental dysfunction plays a critical role, as inadequate invasion of trophoblasts into the uterine arteries results in placental hypoxia. This ischemia triggers the release of factors that exacerbate endothelial dysfunction and systemic hypertension, contributing to the clinical manifestations of PE [27]. The interplay between these factors creates a cycle of worsening vascular health, leading to the characteristic hypertension and proteinuria seen in PE [28].

Preterm delivery, occurring before the completion of 37 weeks of pregnancy, constitutes a major global health concern, being the primary cause of neonatal death and a significant factor in the development of lifelong diseases [29].IR can contribute to Preterm delivery through several interconnected processes. In IR, cells exhibit decreased sensitivity to insulin, leading to elevated blood glucose levels and increased insulin production by the pancreas, a condition known as compensatory hyperinsulinemia [30]. The dysregulation in question is typically associated with the metabolic syndrome, which involves obesity, high blood pressure, and abnormal lipid levels, all recognized as contributors to the risk of experiencing adverse pregnancy outcomes, including Preterm delivery [31].The insulin signaling pathway, which is crucial for glucose uptake and metabolism, becomes disrupted in IR, further exacerbating hyperglycemia [32].Elevated blood glucose can lead to systemic inflammation and oxidative stress, which are detrimental to both maternal and fetal health. These factors can trigger premature labor by affecting uterine contractility and placental function [33]. Thus, the interplay between IR, metabolic syndrome, and hyperglycemia forms a complex pathway that can culminate in Preterm delivery.

The hyperinsulin-normal glucose clamp method, often recognized as the criterion standard for assessing IR, provides exceptional accuracy but faces limitations in large-scale epidemiological investigations owing to its invasive character and the associated high expenses [34]. Consequently, alternative markers such as HOMA-IR and the TyG index have been developed and are increasingly used. These alternatives offer simpler, less invasive, and more cost-effective means of assessing IR, making them suitable for extensive population studies [35].The HOMA-IR index is a widely recognized method for assessing IR; however, its complexity and the high costs associated with fasting insulin measurement limit its practical application in clinical settings [36]. In contrast, the TyG index, which integrates fasting blood sugar and triglyceride levels, offers a more accessible alternative. This index requires fewer laboratory operations and is significantly less expensive, making it more suitable for routine clinical use [37].

Our study discovered a notably positive link between the TyG index during the prenatal period and the subsequent chance of GDM. Correspondingly, Guo et al. have illustrated that this index early in pregnancy serves as a robust harbinger of GDM by the second trimester. An escalation of 2.10 times the risk of GDM was associated with each incremental rise of one unit in the TyG index, notably peaking in the stratum with the highest TyG index relative to the lowest quintile. [38]. A further study carried out in the United States has verified a positive association between the TyG index and GDM, which remains consistent even after considering possible confounding factors. The odds ratio for GDM was 3.43, indicating a significant risk increase with higher TyG index levels [39]. Analysis of a cross-sectional data showed that persons with GDM had much higher TyG index values compared to healthy controls. This suggests that the TyG index may be useful in assessing cardiovascular risk in GDM patients [40]. Within the scope of our study, a noteworthy positive correlation was identified between elevated TyG in early pregnancy and the risk of PE, GH and Preterm delivery. An investigation in Hangzhou, China, revealed a marked correlation between higher TyG index values during the early to mid-pregnancy phase and the augmented risk of Preterm delivery and preeclampsia. Despite controlling for potential confounding variables, the TyG index continued to show an independent link with these outcomes [41].In our subgroup analysis, the association between TyG and PE was not significant in the subgroup with lower education levels. This may be due to unmeasured confounding factors or the potential mediating effect of education level, which obscured the direct association between the TyG index and the risk of PE.However, another study focusing on the role of the TyG index in predicting gestational diabetes and other pregnancy-related complications did not find a significant association with Preterm delivery [38]. Ye et al. [42] also found that there is a positive correlation between increased TyG levels and a greater risk of developing preeclampsia.

Although current literature suggests in analyses that the TyG index correlates with adverse pregnancy outcomes, there are still inconsistent results after adjusting for various factors. The possible reasons may include geographical differences between populations, variations in the gestational weeks arranged for testing this indicator, and differences in the calibration factors used in regression analyses. Future studies must recognize these discrepancies and delve into the possible reasons behind the conflicting outcomes. GDM, PE, GH, and Preterm delivery are prevalent and perilous pregnancy complications, with their underlying mechanisms still unclear. Due to our limited understanding of this pathobiological phenomenon, no effective preventive measures have been established to date. Nonetheless, the TyG index provides a novel and straightforward approach to address these issues, particularly regarding the implementation of the TyG index in screening programs. Therefore, it is recommended to incorporate the TyG index into routine prenatal examinations as a tool for early identification of high-risk pregnancies, to facilitate timely lifestyle interventions, nutritional guidance, or medical interventions.As far as we know, this study is the first to to provide a comprehensive analysis establishing a significant, independent link between the elevated TyG index and the increased risk of GDM, PE, GH and Preterm delivery. This study enrolled 11,942 pregnant women, constituting a substantial sample size that enhances statistical power and significantly contributes to the existing field. The collection of the indicator was in the early stages of pregnancy, and such early detection can lead to timely intervention and management strategies to improve maternal and fetal prognosis. Furthermore, we mitigated potential confounding biases by adjusting for numerous covariates, thereby bolstering the reliability of our findings and extending their applicability to a wider population. Lastly, we utilized sensitivity analysis, subgroup analysis, and interaction analysis to strengthen the reliability of our evaluation of the relationship between the TyG index and the probability of adverse pregnancy outcomes. While our study has several strengths, it is not without potential limitations that warrant consideration. Primarily, although we adjusted for several potential confounding covariates as much as possible, the possibility of residual confounding still exists. Such factors as dietary habits, socioeconomic status, or physical activity may have an impact. Secondly, our study primarily focuses on pregnant women from the Longgang District in Shenzhen, China. Given variations in living environments and dietary habits, the generalizability of our results to other populations may be constrained. Therefore, additional research is necessary to ascertain the universality of our findings. We measured the TyG index only once. The continuous longitudinal changes of this index across different gestational weeks and its correlation with clinical outcomes remain unknown.Finally, the lack of data on fasting insulin levels precludes a direct comparison of the TyG index with other established indices for evaluating IR.

## Conclusion

In conclusion, early pregnancy TyG is robustly and positively correlated with the risk of GDM. An elevated TyG index is significantly and independently associated with heightened risks of PE, GH, and preterm delivery. Furthermore, the findings of our study carry substantial implications for potentially reducing screening expenditures. These results are particularly pertinent to clinical practice and warrant consideration in extensive epidemiological research. Future investigations should examine if interventions aimed at the TyG index could enhance outcomes in pregnancies complicated by adverse events.

## Supplementary Information

Below is the link to the electronic supplementary material.

Supplementary file1 (PDF 71 KB)

Supplementary file2 (PDF 469 KB)

Supplementary file3 (PDF 659 KB)

Supplementary file4 (PDF 691 KB)

Supplementary file5 (DOCX 17 KB)

Supplementary file6 (DOCX 13 KB)

Supplementary file7 (DOCX 13 KB)

Supplementary file8 (DOCX 13 KB)

Supplementary file9 (DOCX 13 KB)

Supplementary file10 (DOCX 13 KB)

Supplementary file11 (DOCX 13 KB)

Supplementary file12 (DOCX 13 KB)

Supplementary file13 (DOCX 13 KB)

## Data Availability

No datasets were generated or analysed during the current study.

## Abbreviations

95% CI: 95% Confidence interval
ACOG: American College of Obstetricians and Gynecologists
ADA: American Diabetes Association
ALB: Albumin
BMI: Body mass index
FPG: Fasting plasma glucose
GDM: Gestational diabetes mellitus
GH: Gestational hypertension
HEC: Hyperinsulinemic-euglycemic clamp
HbA1c: Glycated hemoglobin
HDL-C: High-density lipoprotein cholesterol
IADPSG: Association of Diabetes and Pregnancy Study Groups
IR: Insulin resistance
LDL-C: Low-density lipoprotein cholesterol
OGTT: Oral glucose tolerance test
OR: Odds ratios
PE: Preeclampsia
TG: Triglycerides
TP: Total protein
TyG: Triglyceride-glucose
TC: Total cholesterol

## References

[1] ElSayed NA, Aleppo G, Aroda VR, Bannuru RR, Brown FM, Bruemmer D et al (2023) 2. Classification and diagnosis of diabetes: standards of care in diabetes—2023. Diabetes Care 46(1):S19–S40 36507649 10.2337/dc23-S002 PMC9810477

[2] Burton GJ, Jauniaux E (1997) The human placenta: new perspectives on its formation and function during early pregnancy. Proc Biol Sci 2023(290):20230191 10.1098/rspb.2023.0191 PMC10113033 37072047

[3] Ciaraldi TP, Kettel M, El-Roeiy A, Madar Z, Reichart D, Yen SS et al (1994) Mechanisms of cellular insulin resistance in human pregnancy. Am J Obstet Gynecol 170(2):635–641 8116725 10.1016/s0002-9378(94)70241-1

[4] Catalano PM (2010) Obesity, insulin resistance, and pregnancy outcome. Reproduction 140(3):365–371 20457594 10.1530/REP-10-0088 PMC4179873

[5] Simental-Mendia LE, Rodriguez-Moran M, Guerrero-Romero F (2008) The product of fasting glucose and triglycerides as surrogate for identifying insulin resistance in apparently healthy subjects. Metab Syndr Relat Disord 6(4):299–304 19067533 10.1089/met.2008.0034

[6] Avagimyan A, Pogosova N, Fogacci F, Aghajanova E, Djndoyan Z, Patoulias D et al (2025) Triglyceride-glucose index (TyG) as a novel biomarker in the era of cardiometabolic medicine. Int J Cardiol 418:132663 39426418 10.1016/j.ijcard.2024.132663

[7] Guerrero-Romero F, Simental-Mendia LE, Gonzalez-Ortiz M, Martinez-Abundis E, Ramos-Zavala MG, Hernandez-Gonzalez SO et al (2010) The product of triglycerides and glucose, a simple measure of insulin sensitivity. Comparison with the euglycemic-hyperinsulinemic clamp. J Clin Endocrinol Metab 95(7):3347–3351 20484475 10.1210/jc.2010-0288

[8] Lopez-Jaramillo P, Gomez-Arbelaez D, Martinez-Bello D, Abat MEM, Alhabib KF, Avezum A et al (2023) Association of the triglyceride glucose index as a measure of insulin resistance with mortality and cardiovascular disease in populations from five continents (PURE study): a prospective cohort study. Lancet Healthy Longev 4(1):e23–e33 36521498 10.1016/S2666-7568(22)00247-1

[9] Hong S, Han K, Park CY (2020) The triglyceride glucose index is a simple and low-cost marker associated with atherosclerotic cardiovascular disease: a population-based study. BMC Med 18(1):361 33234146 10.1186/s12916-020-01824-2 PMC7687762

[10] Chiu H, Tsai HJ, Huang JC, Wu PY, Hsu WH, Lee MY et al (2020) Associations between triglyceride-glucose index and micro- and macro-angiopathies in type 2 diabetes mellitus. Nutrients 12(2):328 31991925 10.3390/nu12020328 PMC7071226

[11] Zhang Y, Chu C, Zhong Z, Luo YB, Ning FF, Guo N (2023) High triglyceride-glucose index is associated with poor cardiovascular outcomes in Chinese acute coronary syndrome patients without diabetes mellitus who underwent emergency percutaneous coronary intervention with drug-eluting stents. Front Endocrinol (Lausanne) 14:1101952 36875470 10.3389/fendo.2023.1101952 PMC9975349

[12] Wu S, Xu L, Wu M, Chen S, Wang Y, Tian Y (2021) Association between triglyceride-glucose index and risk of arterial stiffness: a cohort study. Cardiovasc Diabetol 20(1):146 34271940 10.1186/s12933-021-01342-2 PMC8285795

[13] Liu X, Tan Z, Huang Y, Zhao H, Liu M, Yu P et al (2022) Relationship between the triglyceride-glucose index and risk of cardiovascular diseases and mortality in the general population: a systematic review and meta-analysis. Cardiovasc Diabetol 21(1):124 35778731 10.1186/s12933-022-01546-0 PMC9250255

[14] Jiang L, Li AQ (2024) Characteristics and pregnancy outcomes of subtypes of gestational diabetes mellitus based on HOMA-IR and BMI. Arch Gynecol Obstet 310(5):2355–2361 39287682 10.1007/s00404-024-07733-6

[15] Villar J, Cheikh Ismail L, Victora CG, Ohuma EO, Bertino E, Altman DG et al (2014) International standards for newborn weight, length, and head circumference by gestational age and sex: the newborn cross-sectional study of the INTERGROWTH-21st project. Lancet 384(9946):857–868 25209487 10.1016/S0140-6736(14)60932-6

[16] Cornish RP, Magnus MC, Urhoj SK, Santorelli G, Smithers LG, Odd D et al (2024) Maternal pre-pregnancy body mass index and risk of Preterm delivery: a collaboration using large routine health datasets. BMC Med 22(1):10 38178112 10.1186/s12916-023-03230-w PMC10768428

[17] Kantowski T, Schulze Zur Wiesch C, Aberle J, Lautenbach A (2024) Obesity management: sex-specific considerations. Arch Gynecol Obstet 309(5):1745–1752 38329549 10.1007/s00404-023-07367-0 PMC11018683

[18] Zheng H, Chen G, Wu K, Wu W, Huang Z, Wang X et al (2023) Relationship between cumulative exposure to triglyceride-glucose index and heart failure: a prospective cohort study. Cardiovasc Diabetol 22(1):239 37667253 10.1186/s12933-023-01967-5 PMC10476374

[19] Sun YY, Juan J, Xu QQ, Su RN, Hirst JE, Yang HX (2020) Increasing insulin resistance predicts adverse pregnancy outcomes in women with gestational diabetes mellitus. J Diabetes 12(6):438–446 31808991 10.1111/1753-0407.13013

[20] McIntyre HD, Catalano P, Zhang C, Desoye G, Mathiesen ER, Damm P (2019) Gestational diabetes mellitus. Nat Rev Dis Primers 5(1):47 31296866 10.1038/s41572-019-0098-8

[21] Song S, Zhang Y, Qiao X, Duo Y, Xu J, Peng Z et al (2022) HOMA-IR as a risk factor of gestational diabetes mellitus and a novel simple surrogate index in early pregnancy. Int J Gynaecol Obstet 157(3):694–701 34449903 10.1002/ijgo.13905

[22] Plows JF, Stanley JL, Baker PN, Reynolds CM, Vickers MH (2018) The pathophysiology of gestational diabetes mellitus. Int J Mol Sci 19(11):3342 30373146 10.3390/ijms19113342 PMC6274679

[23] Ellerbrock J, Spaanderman B, Drongelen JV, Mulder E, van Lopes Balen V, Schiffer V et al (2022) Role of beta cell function and insulin resistance in the development of gestational diabetes mellitus. Nutrients 14(12):2444 35745174 10.3390/nu14122444 PMC9231208

[24] Steegers EA, von Dadelszen P, Duvekot JJ, Pijnenborg R (2010) Pre-eclampsia. Lancet 376(9741):631–644 20598363 10.1016/S0140-6736(10)60279-6

[25] Roberts JM, Gammill H (2006) Insulin resistance in preeclampsia. Hypertension 47(3):341–342 16446385 10.1161/01.HYP.0000205123.40068.84

[26] Poston L (2006) Endothelial dysfunction in pre-eclampsia. Pharmacol Rep 58(Suppl):69–74 17332674

[27] Tan B, Lin L, Yuan Y, Long Y, Kang Y, Huang B et al (2024) Endothelial progenitor cells control remodeling of uterine spiral arteries for the establishment of utero-placental circulation. Dev Cell 59(14):1842–59.e12 38663400 10.1016/j.devcel.2024.04.009

[28] Agrawal A, Wenger NK (2020) Hypertension during pregnancy. Curr Hypertens Rep 22(9):64 32852628 10.1007/s11906-020-01070-0

[29] The Lancet Child Adolescent Health (2023) The peril and promise of the neonatal period. Lancet Child Adolesc Health 7(12):815 37973249 10.1016/S2352-4642(23)00290-0

[30] Wang X, Yu C, Zhang B, Wang Y (2014) The injurious effects of hyperinsulinism on blood vessels. Cell Biochem Biophys 69(2):213–218 24352639 10.1007/s12013-013-9810-6

[31] Ellerbrock J, Hubers E, Ghossein-Doha C, Schiffer V, Alers RJ, Jorissen L et al (2022) Second-trimester constituents of the metabolic syndrome and pregnancy outcome: an observational cohort study. Nutrients 14(14):2933 35889890 10.3390/nu14142933 PMC9325303

[32] Lake S, Krook A, Zierath JR (2003) Analysis of insulin signaling pathways through comparative genomics. Mapping mechanisms for insulin resistance in type 2 (non-insulin-dependent) diabetes mellitus. Exp Clin Endocrinol Diabetes 111(4):191–197 12845556 10.1055/s-2003-40462

[33] Joo EH, Kim YR, Kim N, Jung JE, Han SH, Cho HY (2021) Effect of endogenic and exogenic oxidative stress triggers on adverse pregnancy outcomes: preeclampsia, fetal growth restriction, gestational diabetes mellitus and preterm delivery. Int J Mol Sci 22(18):10122 34576285 10.3390/ijms221810122 PMC8468091

[34] Robert JJ (1995) Methods for the measurement of insulin resistance hyperinsulinemic euglycemic clamp. Presse Med 24(15):730–734 7784407

[35] Verhulst CEM, Fabricius TW, Teerenstra S, Kristensen PL, Tack CJ, McCrimmon RJ et al (2022) Glycaemic thresholds for counterregulatory hormone and symptom responses to hypoglycaemia in people with and without type 1 diabetes: a systematic review. Diabetologia 65(10):1601–1612 35867127 10.1007/s00125-022-05749-8 PMC9477942

[36] Lupinska A, Aszkielowicz S, Kowalik D, Jeziorny K, Kolasa-Kicinska M, Smalczewska P et al (2024) Comparison of the clinical utility of two insulin resistance indices: IRI-HOMA and IRI-Belfiore in diagnosing insulin resistance and metabolic complications in children based on the results obtained for the Polish population. J Clin Med 13(10):2865 38792408 10.3390/jcm13102865 PMC11122103

[37] Kang B, Yang Y, Lee EY, Yang HK, Kim HS, Lim SY et al (2017) Triglycerides/glucose index is a useful surrogate marker of insulin resistance among adolescents. Int J Obes (Lond) 41(5):789–792 28104918 10.1038/ijo.2017.14

[38] Guo Y, Lu J, Bahani M, Ding G, Wang L, Zhang Y et al (2024) Triglyceride-glucose index in early pregnancy predicts the risk of gestational diabetes: a prospective cohort study. Lipids Health Dis 23(1):87 38528508 10.1186/s12944-024-02076-2 PMC10962154

[39] Zeng Y, Yin L, Yin X, Zhao D (2023) Association of triglyceride-glucose index levels with gestational diabetes mellitus in the US pregnant women: a cross-sectional study. Front Endocrinol (Lausanne) 14:1241372 37881497 10.3389/fendo.2023.1241372 PMC10597685

[40] Ozyildirim S, Barman HA, Dogan O, Ersanli MK, Dogan SM (2023) The relationship between coronary flow reserve and the TyG index in patients with gestational diabetes mellitus. Medicina (Kaunas) 59(10):1811 37893529 10.3390/medicina59101811 PMC10608421

[41] Zhang J, Yin B, Xi Y, Bai Y (2024) Triglyceride-glucose index: a promising biomarker for predicting risks of adverse pregnancy outcomes in Hangzhou, China. Prev Med Rep. 41:102683 38524277 10.1016/j.pmedr.2024.102683 PMC10957496

[42] Ye H, Yin BB, Zhang JH, Xi Y, Chen F, Bai YY (2023) Combining the triglyceride-glucose index and glycated hemoglobin A1c to assess the risk of preeclampsia in women with normal glucose tolerance: a cross-sectional study. Eur Rev Med Pharmacol Sci 27(19):9279–9295 37843342 10.26355/eurrev_202310_33956

