# Supplementary figures and images for: An elevated triglyceride-glucose index in the first-trimester predicts adverse pregnancy outcomes: a retrospective cohort study
Source: Arch Gynecol Obstet. 2025 Feb 26;311(3):915–27. doi: 10.1007/s00404-025-07973-0 (PMC11920334; doi:10.1007/s00404-025-07973-0)

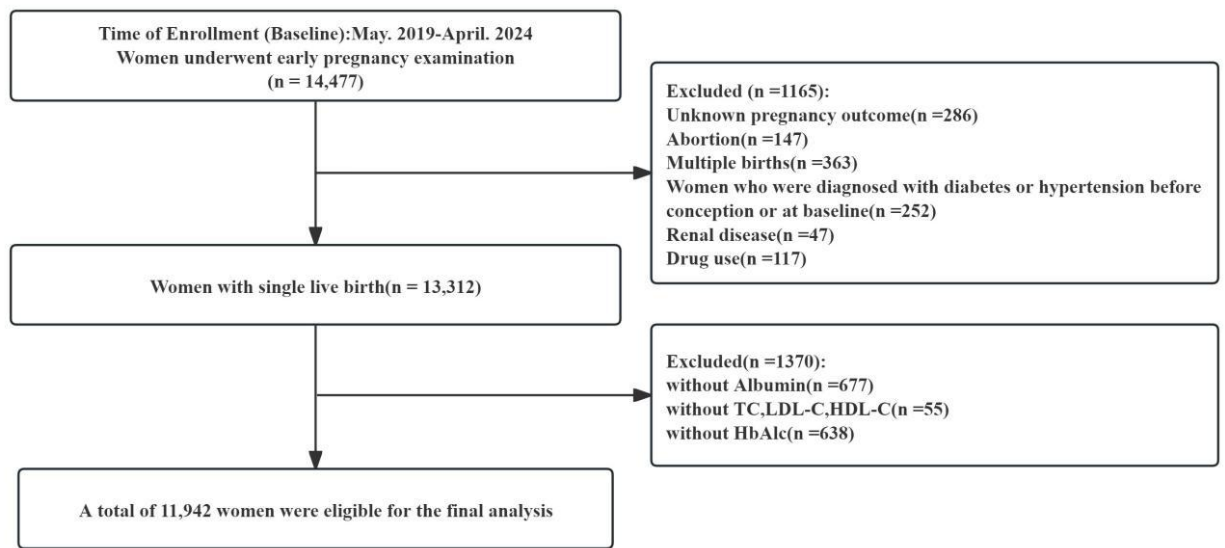

Supplement: Supplementary file 1 — Supplementary file1 (PDF 71 KB) [file 404_2025_7973_MOESM1_ESM.pdf]

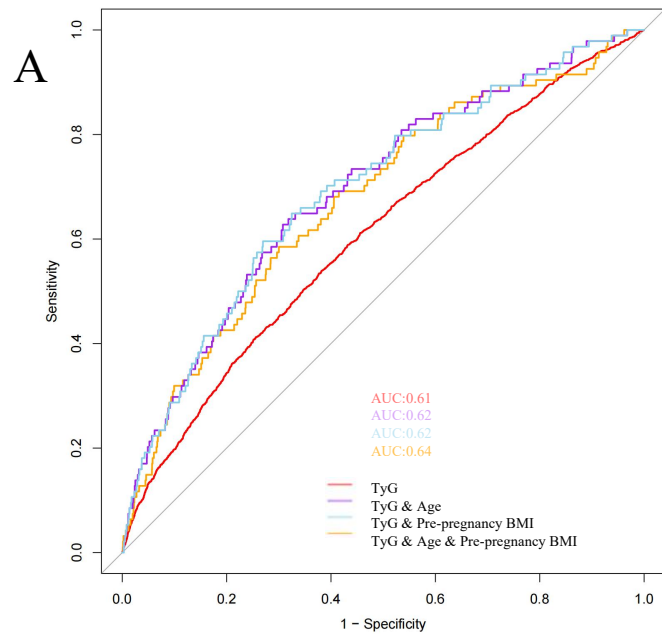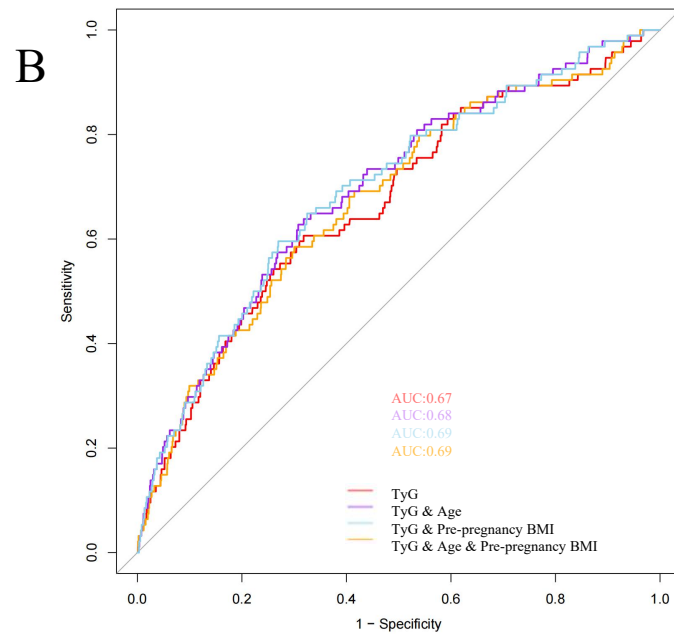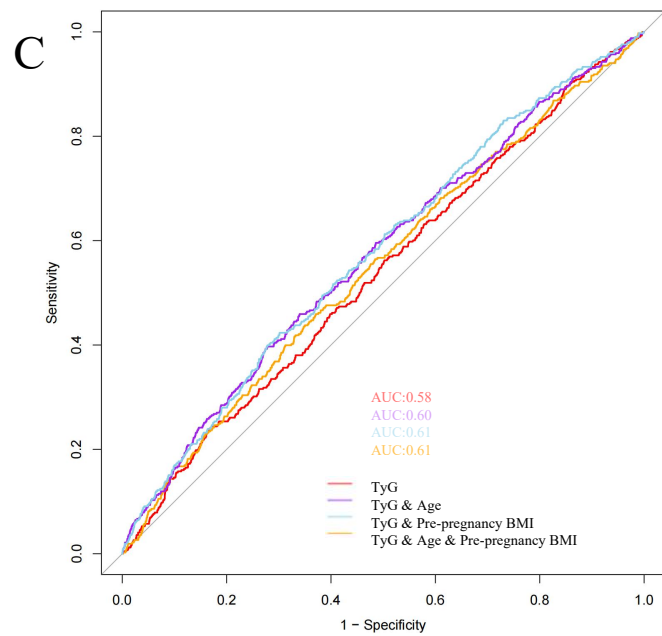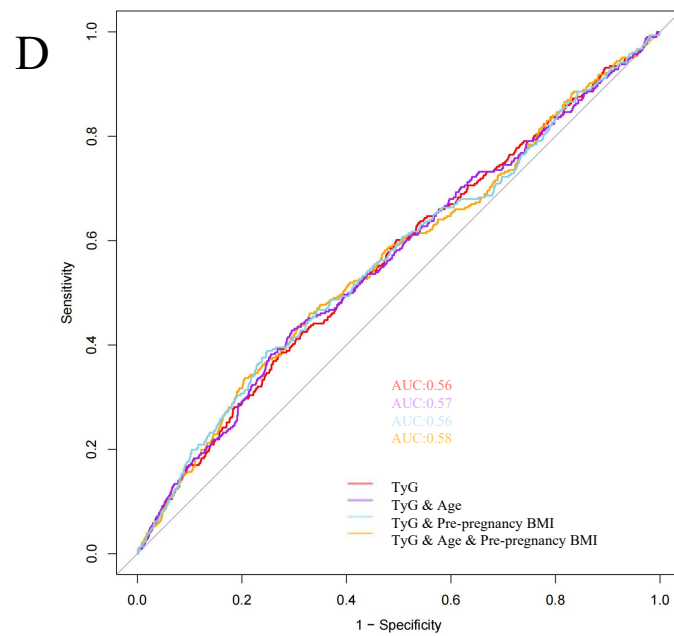

Supplement: Supplementary file 2 — Supplementary file2 (PDF 469 KB) [file 404_2025_7973_MOESM2_ESM.pdf]

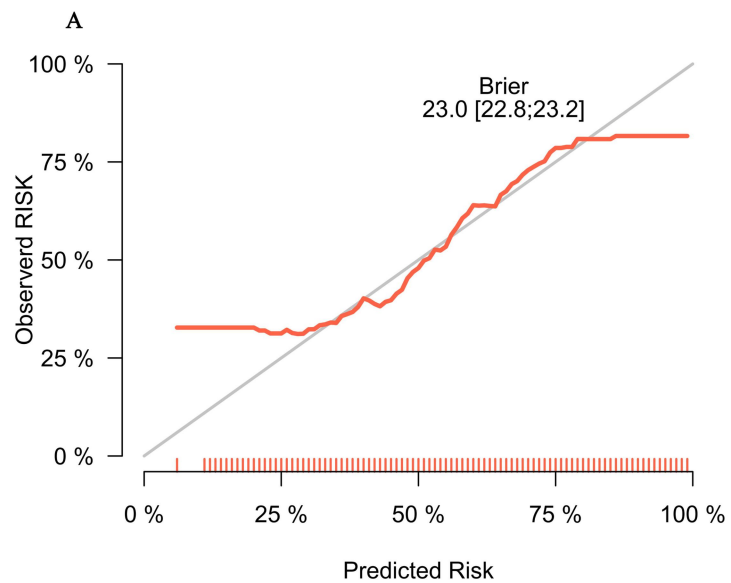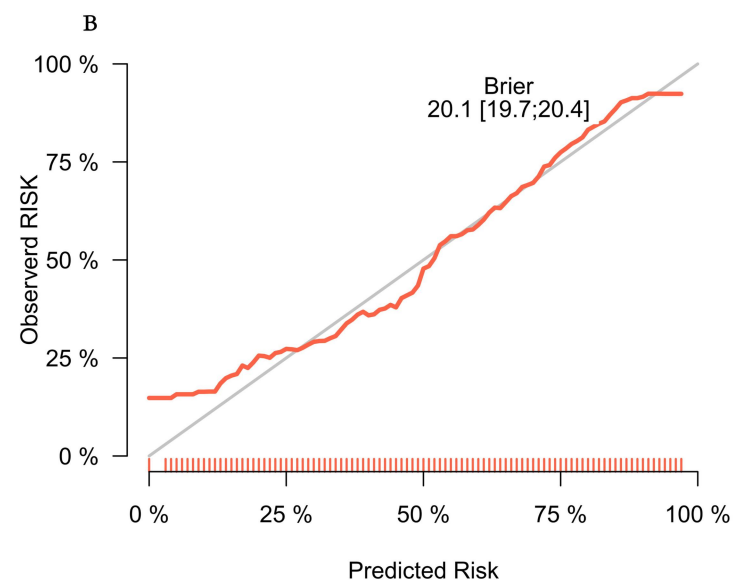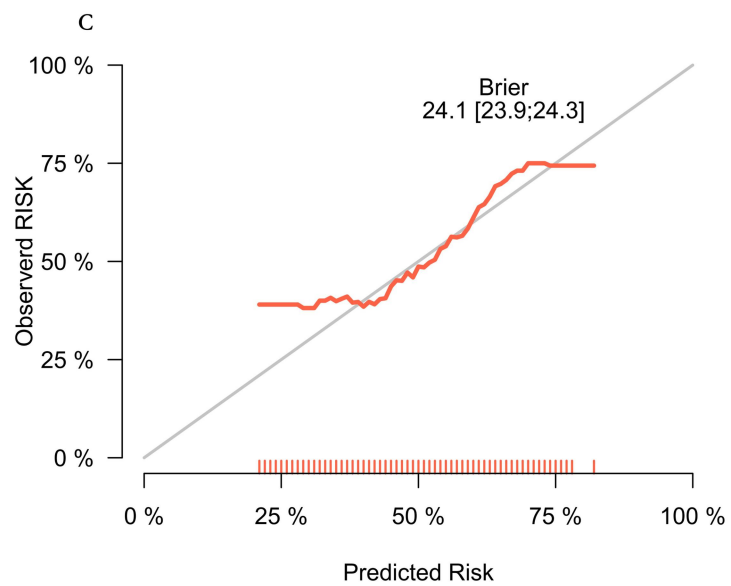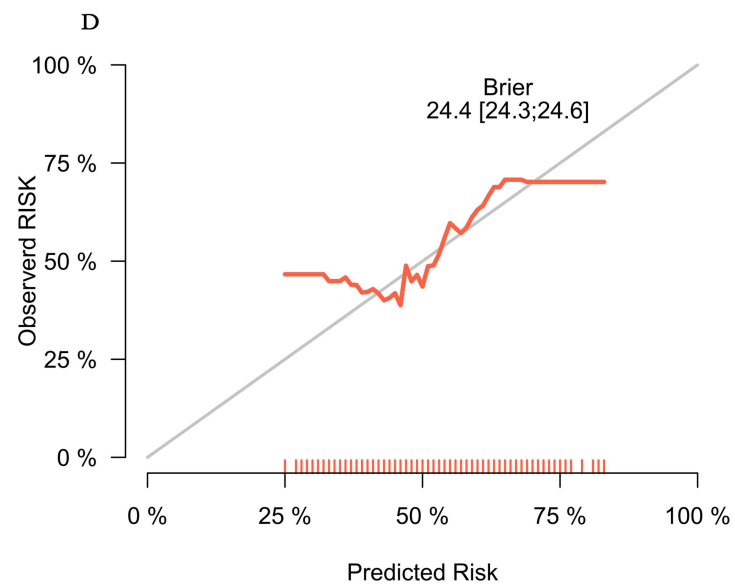

Supplement: Supplementary file 3 — Supplementary file3 (PDF 659 KB) [file 404_2025_7973_MOESM3_ESM.pdf]

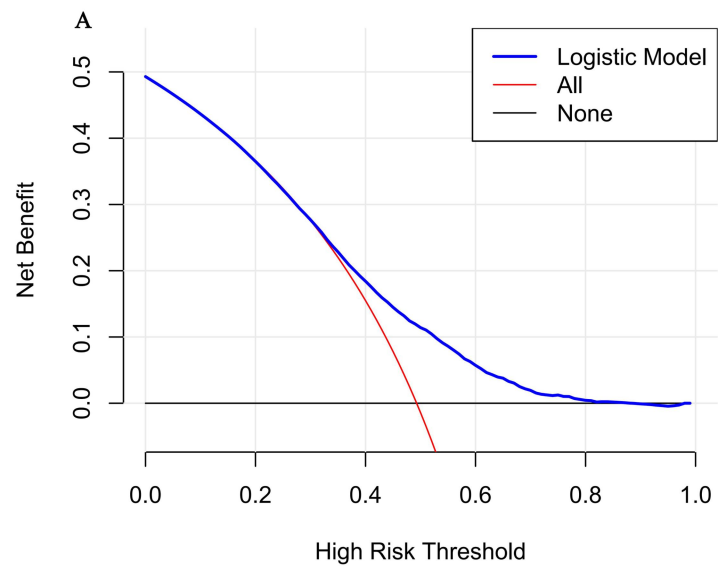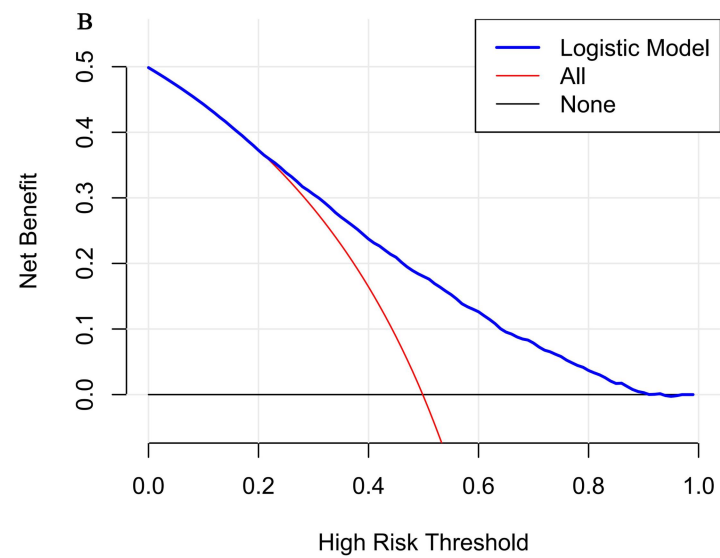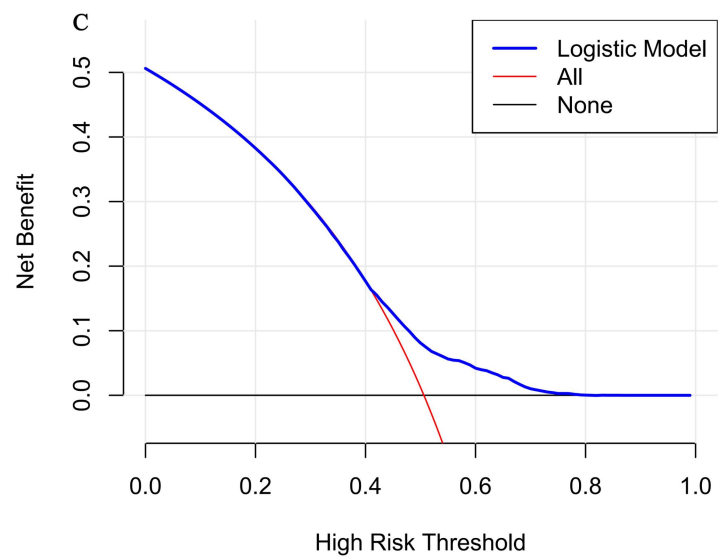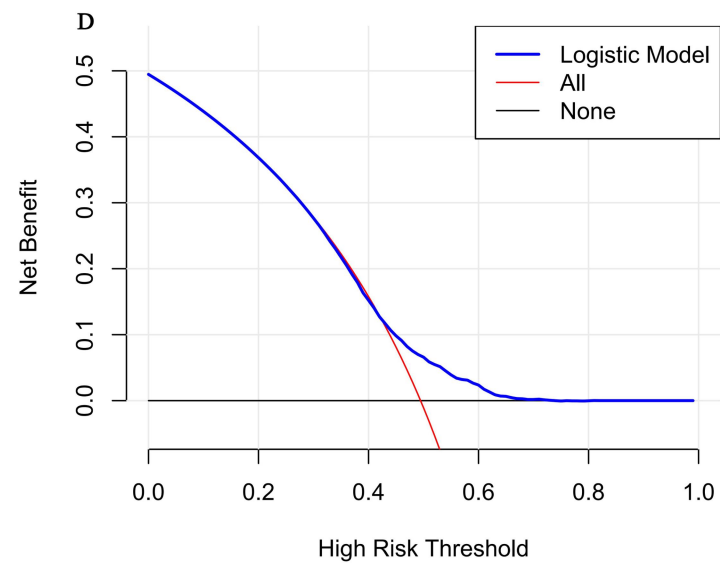

Supplement: Supplementary file 4 — Supplementary file4 (PDF 691 KB) [file 404_2025_7973_MOESM4_ESM.pdf]
