# Supplementary material for: An elevated triglyceride-glucose index in the first-trimester predicts adverse pregnancy outcomes: a retrospective cohort study
Source: Arch Gynecol Obstet. 2025 Feb 26;311(3):915–27. doi: 10.1007/s00404-025-07973-0 (PMC11920334; doi:10.1007/s00404-025-07973-0)
Supplement: Supplementary file 5 — Supplementary file5 (DOCX 17 KB) [file 404_2025_7973_MOESM5_ESM.docx]

**Additional file 1: Table S1 baseline characteristics of the study population by Age**

| **Maternal age** | **Overall** | **≤24** | **25–29** | **30–34** | **≥35** | ***P* value** |
| --- | --- | --- | --- | --- | --- | --- |
| Gestational week at examination, weeks | 9.18 (1.89) | 9.43 (2.05) | 9.17 (1.89) | 9.08 (1.83) | 9.36 (1.92) | **<0.001** |
| Education, years |  |  |  |  |  | **<0.001** |
| 9 | 1302 (10.9) | 197 (24.0) | 514 (11.0) | 392 (8.3) | 199 (11.3) |  |
| 10-12 | 967 (8.1) | 87 (10.6) | 365 (7.8) | 360 (7.7) | 155 (8.8) |  |
| 13-15 | 4907 (41.1) | 445 (54.3) | 2076 (44.5) | 1717 (36.6) | 669 (38.1) |  |
| ≥16 | 4766 (39.9) | 91 (11.1) | 1715 (36.7) | 2228 (47.4) | 732 (41.7) |  |
| SBP, mmHg | 111.25 (11.14) | 110.77 (11.42) | 111.41 (11.03) | 111.03 (11.12) | 111.59 (11.32) | 0.116 |
| DBP, mmHg | 67.22 (8.65) | 66.81 (8.87) | 67.20 (8.46) | 67.26 (8.75) | 67.34 (8.78) | 0.519 |
| Pre-pregnancy BMI, kg/m2 | 21.48 (3.05) | 20.96 (3.37) | 21.11 (3.06) | 21.62 (2.93) | 22.34 (2.99) | **<0.001** |
| Gravidity, n(%) |  |  |  |  |  | **<0.001** |
| 1 | 5072 (42.5) | 544 (66.3) | 2729 (58.4) | 1584 (33.7) | 215 (12.3) |  |
| 2 | 3591 (30.1) | 200 (24.4) | 1269 (27.2) | 1626 (34.6) | 496 (28.3) |  |
| 3 | 1875 (15.7) | 56 (6.8) | 459 (9.8) | 886 (18.9) | 474 (27.0) |  |
| ≥4 | 1404 (11.8) | 20 (2.4) | 213 (4.6) | 601 (12.8) | 570 (32.5) |  |
| Parity, n(%) |  |  |  |  |  | **<0.001** |
| 0 | 6580 (55.1) | 671 (81.8) | 3386 (72.5) | 2155 (45.9) | 368 (21.0) |  |
| 1 | 4234 (35.5) | 136 (16.6) | 1109 (23.7) | 2053 (43.7) | 936 (53.3) |  |
| ≥2 | 1128 (9.4) | 13 (1.6) | 175 (3.7) | 489 (10.4) | 451 (25.7) |  |
| FPG, mg/dL | 84.16 (9.27) | 82.98 (8.45) | 83.53 (8.63) | 84.50 (9.62) | 85.45 (10.12) | **<0.001** |
| TG, mg/dL | 110.91 (49.81) | 103.51 (40.78) | 104.01 (44.70) | 113.34 (50.16) | 126.26 (60.48) | **<0.001** |
| HbAlc, %[mmol/mol] | 4.95 (0.40)[54.10 (4.37)] | 4.86 (0.42)[53.11 (4.59)] | 4.92 (0.36)[53.77 (3.93)] | 4.97 (0.42)[54.32 (4.59)] | 5.04 (0.42)[55.08 (4.59)] | **<0.001** |
| TC, mmol/L | 4.31 (0.69) | 4.22 (0.68) | 4.26 (0.68) | 4.33 (0.69) | 4.42 (0.73) | **<0.001** |
| HDL, mmol/L | 1.52 (0.29) | 1.50 (0.29) | 1.53 (0.29) | 1.52 (0.28) | 1.54 (0.29) | **0.003** |
| LDL, mmol/L | 2.21 (0.56) | 2.18 (0.55) | 2.18 (0.55) | 2.22 (0.57) | 2.28 (0.58) | **<0.001** |
| TP, g/L | 66.12 (3.69) | 66.77 (3.56) | 66.56 (3.64) | 66.00 (3.63) | 64.93 (3.73) | **<0.001** |
| ALB, g/L | 37.94 (2.57) | 38.54 (2.46) | 38.35 (2.51) | 37.85 (2.49) | 36.82 (2.62) | **<0.001** |
| Macrosomia, n(%) | 378 (3.2) | 17 (2.1) | 131 (2.8) | 175 (3.7) | 55 (3.1) | **0.019** |
| Low birth weight, n(%) | 163 (1.4) | 8 (1.0) | 55 (1.2) | 69 (1.5) | 31 (1.8) | 0.202 |
| Preterm delivery, n(%) | 306 (2.6) | 12 (1.5) | 104 (2.2) | 129 (2.7) | 61 (3.5) | **0.006** |
| PE, n(%) | 94 (0.8) | 6 (0.7) | 28 (0.6) | 43 (0.9) | 17 (1.0) | 0.276 |
| GDM, n(%) | 1842 (15.4) | 69 (8.4) | 551 (11.8) | 813 (17.3) | 409 (23.3) | **<0.001** |
| GH, n(%) | 418 (3.5) | 24 (2.9) | 154 (3.3) | 172 (3.7) | 68 (3.9) | 0.483 |
| TyG index | 8.36 (0.41) | 8.29 (0.38) | 8.30 (0.39) | 8.39 (0.40) | 8.50 (0.43) | **<0.001** |
| Categorical data are presented as N (%). Continuous variables are presented as means ± SD  TyG index, triglyceride-glucose index; BMI, body mass index; SBP, systolic blood pressure; DBP, diastolic blood pressure; HbA1c, hemoglobin type A1C; FPG, fasting plasma glucose; TG, triglyceride; HDL, high-density lipoprotein; LDL, low density lipoprotein; TP, Total Protein; ALB, albumin; GDM, gestational diabetes mellitus; GH, Gestational Hypertension; PE, preeclampsia | | | | | | |
