# Supplementary material for: An elevated triglyceride-glucose index in the first-trimester predicts adverse pregnancy outcomes: a retrospective cohort study
Source: Arch Gynecol Obstet. 2025 Feb 26;311(3):915–27. doi: 10.1007/s00404-025-07973-0 (PMC11920334; doi:10.1007/s00404-025-07973-0)
Supplement: Supplementary file 6 — Supplementary file6 (DOCX 13 KB) [file 404_2025_7973_MOESM6_ESM.docx]

**Additional file 1: Table S2** The association between TyG index and the risk of GDM

| **GDM** | **OR (95%CI)** |  |  |
| --- | --- | --- | --- |
|  | **Model 1** | **Model 2** | **Model 3** |
| TyG index (continuous) | 2.61(2.31, 2.94),***P*<0.001** | 2.80(2.46, 3.19),***P*<0.001** | 2.21(1.90, 2.57),***P*<0.001** |
| TyG index (quartiles) |  |  |  |
| Quartile 1 | Reference | Reference |  |
| Quartile 2 | 1.27(1.08, 1.49),***P=*0.003** | 1.29(1.10, 1.51),***P=*0.002** | 1.20(1.02, 1.41),***P=*0.027** |
| Quartile 3 | 1.57(1.35, 1.84),***P*<0.001** | 1.64(1.40, 1.93),***P*<0.001** | 1.44(1.23, 1.70),***P*<0.001** |
| Quartile 4 | 2.61(2.26, 3.03),***P*<0.001** | 2.77(2.38, 3.23),***P*<0.001** | 2.14(1.81, 2.54),***P*<0.001** |
| Bold indicates statistical significance  Model 1: No covariates were adjusted  Model 2: Age, Education, Pre-pregnancy BMI, Gravidity, Parity, gestational week at the examination were adjusted  Model 3: Age, Education, Pre-pregnancy BMI, Gravidity, Parity, gestational week at the examination, SBP, DBP, TC, LDL, HDL,HbAlc, TP, ALB were adjusted  OR odds ratio, 95%CI 95% Confidence Interval, GDM gestational diabetes mellitus | | | |
