# Supplementary material for: An elevated triglyceride-glucose index in the first-trimester predicts adverse pregnancy outcomes: a retrospective cohort study
Source: Arch Gynecol Obstet. 2025 Feb 26;311(3):915–27. doi: 10.1007/s00404-025-07973-0 (PMC11920334; doi:10.1007/s00404-025-07973-0)
Supplement: Supplementary file 7 — Supplementary file7 (DOCX 13 KB) [file 404_2025_7973_MOESM7_ESM.docx]

**Additional file 1: Table S3** The association between TyG index and the risk of PE

| **PE** | **OR (95%CI)** |  |  |
| --- | --- | --- | --- |
|  | **Model 1** | **Model 2** | **Model 3** |
| TyG index (continuous) | 3.98(2.58, 6.05),***P*<0.001** | 4.42(2.79, 6.88),***P*<0.001** | 2.53(1.43, 4.44),***P*<0.001** |
| TyG index (quartiles) |  |  |  |
| Quartile 1 | Reference | Reference |  |
| Quartile 2 | 1.50(0.68, 3.46),*P=*0.319 | 1.53(0.69, 3.54),*P=*0.298 | 1.25(0.56, 2.89),*P=*0.591 |
| Quartile 3 | 2.01(0.96, 4.48),*P=*0.073 | 2.14(1.01, 4.81),*P=*0.053 | 1.46(0.68, 3.34),*P=*0.341 |
| Quartile 4 | 4.97(2.62, 10.4),***P*<0.001** | 5.48(2.81, 11.7),***P*<0.001** | 2.89(1.39, 6.50),***P=*0.007** |
| Bold indicates statistical significance  Model 1: No covariates were adjusted  Model 2: Age, Education, Pre-pregnancy BMI, Gravidity, Parity, gestational week at the examination were adjusted  Model 3: Age, Education, Pre-pregnancy BMI, Gravidity, Parity, gestational week at the examination, SBP, DBP, TC, LDL, HDL,HbAlc, TP, ALB were adjusted  OR odds ratio, 95%CI 95% Confidence Interval, PE preeclampsia | | | |
