# Supplementary material for: An elevated triglyceride-glucose index in the first-trimester predicts adverse pregnancy outcomes: a retrospective cohort study
Source: Arch Gynecol Obstet. 2025 Feb 26;311(3):915–27. doi: 10.1007/s00404-025-07973-0 (PMC11920334; doi:10.1007/s00404-025-07973-0)
Supplement: Supplementary file 8 — Supplementary file8 (DOCX 13 KB) [file 404_2025_7973_MOESM8_ESM.docx]

**Additional file 1: Table S4** The association between TyG index and the risk of GH

| **GH** | **OR (95%CI)** |  |  |
| --- | --- | --- | --- |
|  | **Model 1** | **Model 2** | **Model 3** |
| TyG index (continuous) | 1.38(1.09, 1.75),***P*<0.001** | 1.49(1.15, 1.91),***P=*0.002** | 1.49(1.12, 1.97),***P=*0.006** |
| TyG index (quartiles) |  |  |  |
| Quartile 1 | Reference | Reference |  |
| Quartile 2 | 1.05(0.78, 1.40),*P=*0.764 | 1.06(0.79, 1.43),*P=*0.680 | 1.07(0.80, 1.44),*P=*0.652 |
| Quartile 3 | 1.19(0.90, 1.59),*P=*0.221 | 1.25(0.94, 1.68),*P=*0.133 | 1.26(0.93, 1.69),*P=*0.135 |
| Quartile 4 | 1.38(1.05, 1.82),***P=*0.022** | 1.48(1.10, 1.98),***P=*0.009** | 1.47(1.07, 2.02),***P=*0.019** |
| Bold indicates statistical significance  Model 1: No covariates were adjusted  Model 2: Age, Education, Pre-pregnancy BMI, Gravidity, Parity, gestational week at the examination were adjusted  Model 3: Age, Education, Pre-pregnancy BMI, Gravidity, Parity, gestational week at the examination, SBP, DBP, TC, LDL, HDL, HbAlc, TP, ALB were adjusted  OR odds ratio, 95%CI 95% Confidence Interval, GH Gestational Hypertension | | | |
