# Supplementary material for: An elevated triglyceride-glucose index in the first-trimester predicts adverse pregnancy outcomes: a retrospective cohort study
Source: Arch Gynecol Obstet. 2025 Feb 26;311(3):915–27. doi: 10.1007/s00404-025-07973-0 (PMC11920334; doi:10.1007/s00404-025-07973-0)
Supplement: Supplementary file 9 — Supplementary file9 (DOCX 13 KB) [file 404_2025_7973_MOESM9_ESM.docx]

**Additional file 1: Table S5**  The association between TyG index and the risk of Preterm delivery

| **Preterm delivery** | **OR (95%CI)** |  |  |
| --- | --- | --- | --- |
|  | **Model 1** | **Model 2** | **Model 3** |
| TyG index (continuous) | 1.72(1.31, 2.24),***P*<0.001** | 1.72(1.29, 2.29),***P*<0.001** | 1.78(1.27, 2.48),***P*<0.001** |
| TyG index (quartiles) |  |  |  |
| Quartile 1 | Reference | Reference |  |
| Quartile 2 | 0.90(0.63, 1.30),*P=*0.585 | 0.90(0.63, 1.29),*P=*0.568 | 0.91(0.63, 1.31),*P=*0.610 |
| Quartile 3 | 1.22(0.88, 1.72),*P=*0.235 | 1.24(0.88, 1.75),*P=*0.227 | 1.26(0.88, 1.79),*P=*0.207 |
| Quartile 4 | 1.68(1.23, 2.31),***P*<0.001** | 1.69(1.21, 2.37),***P=*0.002** | 1.75(1.21, 2.56),***P=*0.003** |
| Bold indicates statistical significance  Model 1: No covariates were adjusted  Model 2: Age, Education, Pre-pregnancy BMI, Gravidity, Parity, gestational week at the examination were adjusted  Model 3: Age, Education, Pre-pregnancy BMI, Gravidity, Parity, gestational week at the examination, SBP, DBP, TC, LDL, HDL, HbAlc, TP, ALB were adjusted  OR odds ratio, 95%CI 95% Confidence Interval | | | |
