# Supplementary material for: An elevated triglyceride-glucose index in the first-trimester predicts adverse pregnancy outcomes: a retrospective cohort study
Source: Arch Gynecol Obstet. 2025 Feb 26;311(3):915–27. doi: 10.1007/s00404-025-07973-0 (PMC11920334; doi:10.1007/s00404-025-07973-0)
Supplement: Supplementary file 10 — Supplementary file10 (DOCX 13 KB) [file 404_2025_7973_MOESM10_ESM.docx]

**Additional file 1: Table S6** The association between TyG index and the risk of Macrosomia

| **Macrosomia** | **OR (95%CI)** |  |  |
| --- | --- | --- | --- |
|  | **Model 1** | **Model 2** | **Model 3** |
| TyG index (continuous) | 1.26(0.98, 1.61),***P*<0.001** | 1.30(1.00, 1.70),*P=*0.050 | 1.33(0.98, 1.79),*P=*0.067 |
| TyG index (quartiles) |  |  |  |
| Quartile 1 | Reference | Reference |  |
| Quartile 2 | 0.82(0.61, 1.12),*P=*0.215 | 0.83(0.61, 1.12),*P=*0.226 | 0.83(0.61, 1.14),*P=*0.253 |
| Quartile 3 | 1.08(0.81, 1.44),*P=*0.609 | 1.10(0.82, 1.48),*P=*0.515 | 1.11(0.82, 1.51),*P=*0.491 |
| Quartile 4 | 1.17(0.88, 1.55),*P=*0.281 | 1.21(0.89, 1.63),*P=*0.221 | 1.22(0.87, 1.70),*P=*0.246 |
| Bold indicates statistical significance  Model 1: No covariates were adjusted  Model 2: Age, Education, Pre-pregnancy BMI, Gravidity, Parity, gestational week at the examination were adjusted  Model 3: Age, Education, Pre-pregnancy BMI, Gravidity, Parity, gestational week at the examination, SBP, DBP, TC, LDL, HDL, HbAlc, TP, ALB were adjusted  OR odds ratio, 95%CI 95% Confidence Interval | | | |
