# Supplementary material for: An elevated triglyceride-glucose index in the first-trimester predicts adverse pregnancy outcomes: a retrospective cohort study
Source: Arch Gynecol Obstet. 2025 Feb 26;311(3):915–27. doi: 10.1007/s00404-025-07973-0 (PMC11920334; doi:10.1007/s00404-025-07973-0)
Supplement: Supplementary file 11 — Supplementary file11 (DOCX 13 KB) [file 404_2025_7973_MOESM11_ESM.docx]

**Additional file 1: Table S7**  The association between TyG index and the risk of Low birth weight

| **Low birth weight** | **OR (95%CI)** |  |  |
| --- | --- | --- | --- |
|  | **Model 1** | **Model 2** | **Model 3** |
| TyG index (continuous) | 1.49(1.03, 2.15),***P=*0.032** | 1.45(0.97, 2.13),*P=*0.066 | 1.54(0.97, 2.43),*P=*0.065 |
| TyG index (quartiles) |  |  |  |
| Quartile 1 | Reference | Reference |  |
| Quartile 2 | 1.26(0.79, 2.04),*P=*0.336 | 1.24(0.77, 2.01),*P=*0.374 | 1.26(0.78, 2.05),*P=*0.348 |
| Quartile 3 | 1.33(0.83, 2.14),*P=*0.237 | 1.30(0.80, 2.11),*P=*0.289 | 1.32(0.81, 2.19),*P=*0.265 |
| Quartile 4 | 1.69(1.09, 2.67),***P=*0.022** | 1.63(1.02, 2.64),***P=*0.042** | 1.74(1.04, 2.95),***P=*0.037** |
| Bold indicates statistical significance  Model 1: No covariates were adjusted  Model 2: Age, Education, Pre-pregnancy BMI, Gravidity, Parity, gestational week at the examination were adjusted  Model 3: Age, Education, Pre-pregnancy BMI, Gravidity, Parity, gestational week at the examination, SBP, DBP, TC, LDL, HDL, HbAlc, TP, ALB were adjusted  OR odds ratio, 95%CI 95% Confidence Interval | | | |
