# Supplementary material for: An elevated triglyceride-glucose index in the first-trimester predicts adverse pregnancy outcomes: a retrospective cohort study
Source: Arch Gynecol Obstet. 2025 Feb 26;311(3):915–27. doi: 10.1007/s00404-025-07973-0 (PMC11920334; doi:10.1007/s00404-025-07973-0)
Supplement: Supplementary file 12 — Supplementary file12 (DOCX 13 KB) [file 404_2025_7973_MOESM12_ESM.docx]

**Additional file 2: Table S2 The association of GDM, PE, GH, Preterm delivery,** **Macrosomia, and Low birth weight on TyG index levels**

| TyG index | β (95%CI) |  |  |
| --- | --- | --- | --- |
|  | Model 1 | Model 2 | Model 3 |
| GDM | 0.163 (0.143,0.183), **p<0.0001** | 0.144 (0.125,0.163),  **p<0.0001** | 0.082 (0.064,0.097),  **p=0.02** |
| PE | 0.265 (0.183,0.347),  **p=0.01** | 0.246 (0.17,0.323),  **p=0.005** | 0.113 (0.043,0.178),  **p=0.0013** |
| GH | 0.055 (0.015,0.095), **p=0.0065** | 0.058 (0.021,0.095),  **p=0.002** | 0.0182 (-0.014,0.05),  p=0.2732 |
| Preterm delivery | 0.093 (0.047,0.139),  **p<0.001** | 0.082 (0.039,0.125),  **p<0.001** | 0.074(0.032,0.107),  **p<0.001** |
| Macrosomia | 0.038 (-0.003,0.08),  p=0.0701 | 0.039 (0,0.077),  p=0.0506 | 0.038 (-0.003,0.08),  p=0.1914 |
| Low birth weight | 0.069 (0.006,0.131), **p=0.0323** | 0.046 (-0.012,0.105),  p=0.1195 | 0.048 (-0.003,0.1),  p=0.0625 |
| Bold indicates statistical significance  Model 1: No covariates were adjusted  Model 2: Age, Education, Pre-pregnancy BMI, Gravidity, Parity, gestational week at the examination were adjusted  Model 3: Age, Education, Pre-pregnancy BMI, Gravidity, Parityy, gestational week at the examination, SBP, DBP, TC, LDL, HDL, HbAlc, TP, ALB were adjusted  95%CI 95% Confidence Interval, TyG index triglyceride-glucose index, GDM gestational diabetes mellitus, GH Gestational Hypertension, PE preeclampsia | | | |
