# Supplementary material for: An elevated triglyceride-glucose index in the first-trimester predicts adverse pregnancy outcomes: a retrospective cohort study
Source: Arch Gynecol Obstet. 2025 Feb 26;311(3):915–27. doi: 10.1007/s00404-025-07973-0 (PMC11920334; doi:10.1007/s00404-025-07973-0)
Supplement: Supplementary file 13 — Supplementary file13 (DOCX 13 KB) [file 404_2025_7973_MOESM13_ESM.docx]

**Additional file 3: Table S3 The association of GDM, PE and Preterm delivery on TyG index levels in different age subgroup.**

| TyG index | β(95%CI) | P for interaction |
| --- | --- | --- |
| **GDM** |  |  |
| **Age** |  | 0.4563 |
| ≤24 | 0.158 (0.128, 0.188), **p<0.001** |  |
| 25–29 | 0.134 (0.100, 0.168), **p<0.001** |  |
| 30–34 | 0.094 (0.002, 0.186), **p=0.045** |  |
| ≥35 | 0.129 (0.083, 0.175), **p<0.001** |  |
| **PE** |  |  |
| **Age** |  | 0.646 |
| ≤24 | 0.355 (0.234, 0.476), **p<0.001** |  |
| 25–29 | 0.123 (-0.021, 0.268), p=0.094 |  |
| 30–34 | 0.206 (-0.096, 0.508), p=0.180 |  |
| ≥35 | 0.224 (0.020, 0.428), **p=0.031** |  |
| **Preterm delivery** |  |  |
| **Age** |  | 0.442 |
| ≤24 | 0.122 (0.052, 0.193), **p<0.001** |  |
| 25–29 | 0.083 (0.007, 0.158), **p=0.032** |  |
| 30–34 | -0.012 (-0.226, 0.202), p=0.914 |  |
| ≥35 | 0.003 (-0.106, 0.112), p=0.959 |  |
| 95%CI 95% Confidence Interval, TyG index triglyceride-glucose index, GDM gestational diabetes mellitus, PE preeclampsia | | |
